# Meta-analysis of the association between nut consumption and the risks of cancer incidence and cancer-specific mortality

**DOI:** 10.18632/aging.103292

**Published:** 2020-06-02

**Authors:** Dai Zhang, Cong Dai, Linghui Zhou, Yiche Li, Kang Liu, Yu-Jiao Deng, Na Li, Yi Zheng, Qian Hao, Si Yang, Dingli Song, Ying Wu, Zhen Zhai, Shiyi Cao, Zhijun Dai

**Affiliations:** 1 Department of Breast Surgery, The First Affiliated Hospital, College of Medicine, Zhejiang University, Hangzhou, China; 2 Department of Oncology, The Second Affiliated Hospital of Xi’an Jiaotong University, Xi’an, China; 3 Department of Thyroid and Breast Surgery, Xi 'an International Medical Center Hospital, Xi'an, China; 4 Breast Center Department, The Fourth Hospital of Hebei Medical University, Hebei Medical University, Shijiazhuang, China; 5 Department of Hepatobiliary Surgery, The First Affiliated Hospital of Xi'an Jiaotong University, Xi'an, China; 6 School of Public Health, Tongji Medical College, Huazhong University of Science and Technology, Wuhan, China

**Keywords:** cancer, risk, mortality, nuts, meta-analysis

## Abstract

Previous studies have indicated a correlation between nut intake and cancer risk in humans. This meta-analysis aimed to determine the relationship between nut consumption and the risks of cancer incidence and mortality. The PubMed, Embase, and Web of Science databases were searched up to August 2019. Relative risks and 95% confidence intervals were calculated using random-effects and fixed-effects models. We included 38 studies on nut consumption and cancer risk and 9 studies on cancer-specific mortality. Compared with no nut intake, nut intake was associated with a lower cancer risk (Relative Risk=0.90; 95% confidence interval, 0.86–0.94). Inverse associations were observed with colorectal cancer, gastric cancer, pancreatic cancer, and lung cancer in subgroup analyses. Tree nut consumption was found to reduce cancer risk (Relative Risk=0.88; 95% confidence interval, 0.79–0.99). Dose-response curves suggested that protective benefits against cancer increased with increased nut intake (P=0.005, P-nonlinearity=0.0414). An inverse correlation with cancer-specific mortality (Odd Ratio=0.90; 95% confidence interval, 0.88–0.92) was observed. In conclusion, nut consumption is inversely associated with the risks of cancer incidence and mortality; a higher intake is significantly associated with a lower cancer risk.

## INTRODUCTION

Cancer is a major public health challenge worldwide. Because of the rapid growth and aging of the world population, cancer is predicted to be the leading cause of death globally in the 21^st^ century. Moreover, cancer is expected to be the predominant challenge to an increasing life expectancy. Global Cancer Statistics 2018 estimated that 18.1 million new cancer cases and 9.6 million cancer deaths occurred globally in 2018 [1]. An estimated 606,880 cancer deaths and 1,762,450 cancer diagnoses occurred in the US in 2019 [2]. Given the rapid growth of global cancer morbidity and mortality rates, cancer prevention has significant implications for reducing the global health burden.

There is growing interest in nuts and their health benefits. Results from several epidemiological studies have revealed beneficial effects of nut consumption on health outcomes [3, 4]. Nuts are available worldwide, rich in nutrients, and composed of a hard shell with an edible kernel. Almonds, hazelnuts, cashew nuts, Brazil nuts, macadamias, walnuts, and pistachios are classified as tree nuts. Nutrient-dense tree nuts as well as peanuts have unique nutritional functions. Nuts are included in most human diets; are rich in nutrients such as proteins, monounsaturated and polyunsaturated fatty acids including long-chain n-3 fatty acids, fiber, antioxidants, and several other bioactive compounds; and have proven beneficial health effects [5]. Relevant epidemiological studies have suggested that nuts, because of their protective effects on health, are essential components in healthy and balanced diets. Furthermore, the beneficial effects of nuts on chronic diseases [5–10] have been confirmed. As expected, recent epidemiological studies identified an inverse correlation between frequent nut consumption and the risks of cancer incidence [4] and mortality [8]. However, other studies observed no significant association [11, 12]. Therefore, these conclusions require further investigation. Additionally, there is insufficient evidence on the association between specific nut types and different cancer types [4, 8, 13, 14]. Furthermore, current studies show no evidence of the association between nut intake and cancer risk from a dose-response analysis. Moreover, no exclusive meta-analyses have been conducted to assess the correlation between nut consumption and the risks of cancer incidence and mortality, while previous meta-analyses included other diseases such as strokes, diabetes, and cardiovascular diseases [4, 8, 15]. Hence, the current meta-analysis aimed to determine the relationship between nut consumption and the risks of cancer incidence and mortality and provide an updated and comprehensive assessment using available studies.

## RESULTS

### Literature search

**Figure 1:**
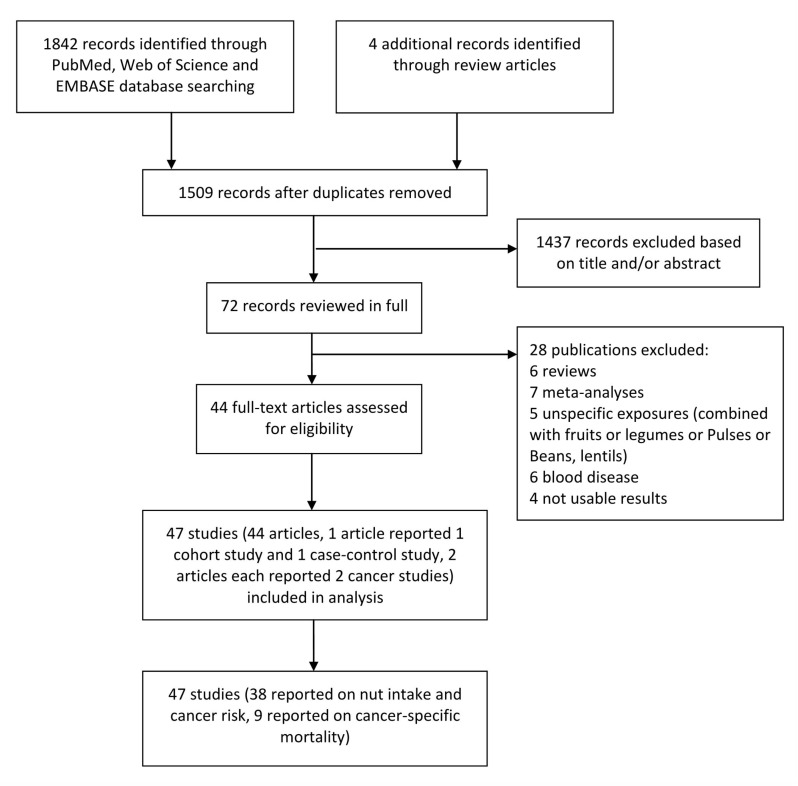
**The process of identification and inclusion of studies.**

Figure 1 shows the overall search process and the study selection and inclusion processes. A total of 1842 studies were identified by searching the databases, while 4 additional studies were identified through relevant reviews and meta-analyses. After the title and abstract review and exclusion of duplicates, 72 studies were reviewed in full. After excluding 28 unqualified articles, we included 38 studies (35 publications, one publication reporting 1 cohort study and 1 case-control study at the same time, and 2 publications reporting 2 different cancer studies each) on nut consumption and cancer risk, including 22 cohort studies and 16 case-control studies. Nine studies were selected for the analysis of the association between nut consumption and cancer-specific mortality.

### Characteristics of the included studies

The characteristics of the included 38 studies are presented in Supplementary Table 1. These selected studies were published between 1985 and 2019. Among the studies on cancer risk, 28 were from developed countries (USA, Sweden, Japan, Canada, Korea, Australia, Greece, and Netherlands) and the remaining 10 studies were from developing countries (Iran, Jamaica, Egypt, Iran, and China). The analysis involved 10 cancer types. The study participants were mainly adults. Dose-response analyses of nut intake and cancer risk were performed in 16 cohort studies (12 publications; 1 reporting 4 sub-studies; and 1 reporting 2 sub-studies). Eleven studies exclusively included men, while 16 studies were conducted exclusively in women. 32 studies recorded the mean age of the study population at baseline, while 6 studies provided age in groups. Supplementary Table 2 shows the characteristics of the 9 cohort studies on cancer-specific mortality. All included studies reported estimations after the adjustment for covariates. Detailed quality scores for each study are listed in Supplementary Tables 1, 2. Moreover, dietary data in most of the included studies were collected using a validated 62-item Food Frequency Questionnaire developed by Wadolowska and Niedzwiedzka [16]. Overall, the included studies (31 cohort studies, 16 case-control studies) were of high quality. The quality evaluation score of the former ranged from 7 to 9 points, while that of the latter ranged from 6 to 9. The average overall quality score was 7.8.

### Nut intake and the risk of total cancer

**Figure 2:**
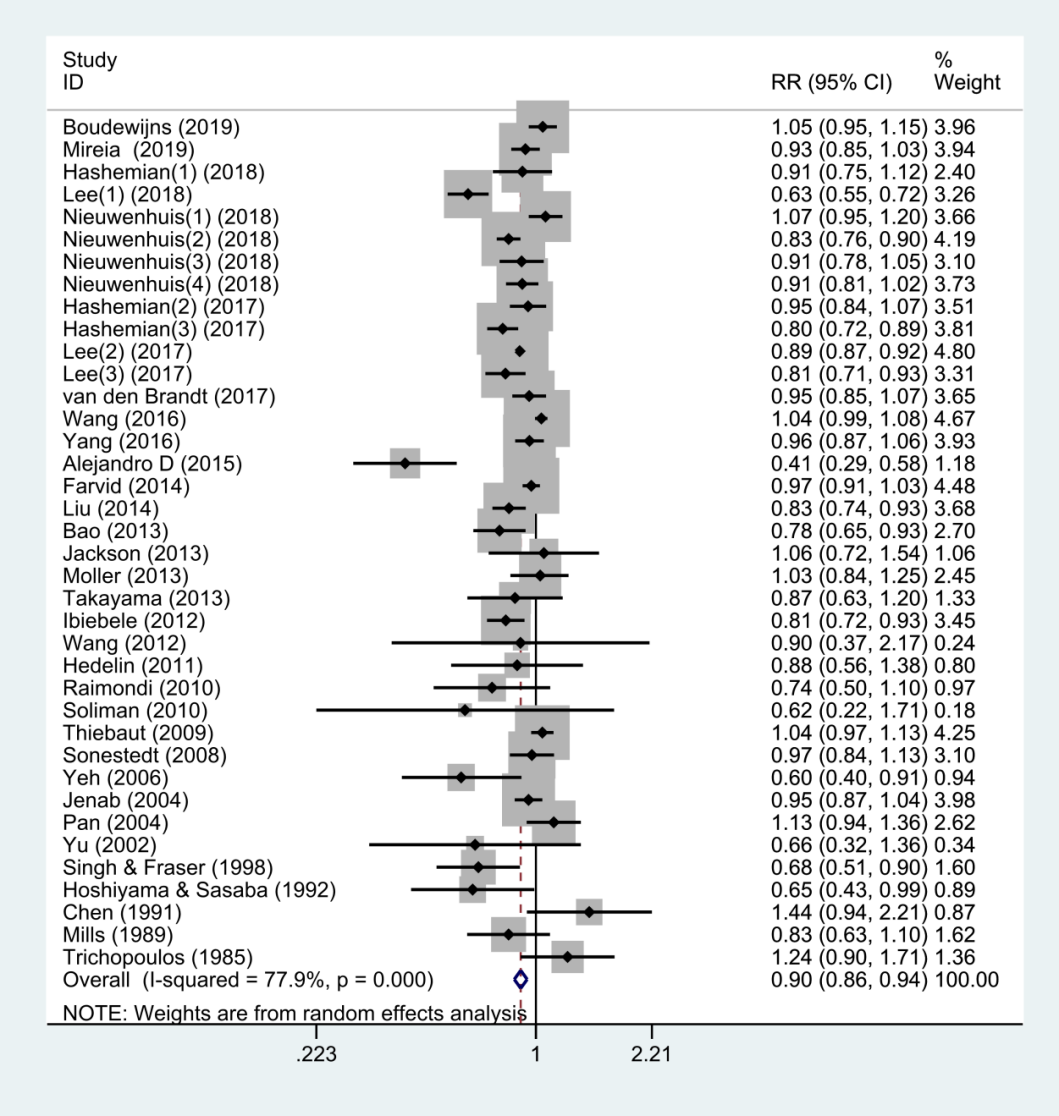
**Overall meta-analyses of the association between nut intake and the risk of cancer.** Note: Weights are from the random-effects analysis. Abbreviations: RR, relative risk; CI, confidence interval.

The results of the meta-analysis of the association between nut intake and total cancer risk calculated by the random-effects model are presented in Figure 2. The pooled estimations of 38 studies identified an overall negative correlation (RR = 0.90; 95% CI, 0.86–0.94; *P* < 0.001) between nut intake and no nut intake with existing evidence of heterogeneity (*P* < 0.001, I^2^ = 77.9%). Meta regression analysis was also performed according to study quality scores and adjustment for age, and the estimated values of regression coefficients were -0.025 and -0.003, respectively. And both the results showed that the study quality (τ^2^ = 0.017, *P* = 0.540) and age (τ^2^ = 0.009, *P* = 0.368) were not the sources of the heterogeneity. Results from the sensitivity analysis indicated that after the exclusion of individual studies from the analysis one by one, the overall results of the analysis of total cancer remained consistent (Supplementary Figure 1). Given the large study span, we also conducted a sensitivity analysis by year (newer studies published after 2010, older studies published in or before 2010); the results remained consistent (Supplementary Figures 2, 3). Furthermore, no obvious asymmetry was observed in the funnel plots (Supplementary Figure 4) for nut consumption and cancer risk. We obtained values of *P* = 0.203 on Egger’s linear regression test and *P* = 0.294 on Begg’s test. Therefore, we found no publication bias in our study.

### Subgroup analyses

The results of subgroup analy1ses stratified by study design, sex, cancer type, nut category, and socioeconomic status are shown in Table 1. No significant difference was observed among the cohort studies (RR = 0.93; 95% CI, 0.89–0.97; *P* = 0.001) and the case-control studies (RR = 0.84; 95% CI, 0.74–0.96; *P* = 0.012) for overall cancer (Supplementary Figure 5).

**Table 1:** Subgroup analyses of nut consumption and cancer risk.

	**Number of studies**	**Results**			**Heterogeneity**	
		**RR**	**95%CI**	**P value**	**I^2^ (%)**	**P value**
All	38	0.90	(0.86-0.94)	<0.001	77.9	<0.001
Subgroup						
Study design						
Cohort	22	0.93	(0.89-0.97)	0.001	74.9	<0.001
Case-control	16	0.84	(0.74-0.96)	0.012	77.5	<0.001
Gender						
Female	16	0.87	(0.79-0.95)	0.001	81.8	<0.001
Male	11	0.92	(0.84-1.01)	0.08	73.1	<0.001
Socioeconomic status						
Developed country	28	0.90	(0.86-0.94)	<0.001	80.5	<0.001
Developing country	10	0.90	(0.72-1.13)	0.365	42	0.111
Cancer type						
Breast cancer	6	0.90	(0.80-1.01)	0.067	85	<0.001
Esophagus cancer	3	0.97	(0.88-1.06)	0.498	0	0.427
Gastric cancer	5	0.83	(0.71-0.97)	0.017	54.4	0.067
Colorectal cancer	5	0.77	(0.63-0.94)	0.011	88.2	<0.001
Prostate cancer	6	1.03	(0.99-1.07)	0.139	2.2	0.402
Pancreatic cancer	3	0.89	(0.81-0.98)	0.015	31.3	0.231
Lung cancer	3	0.89	(0.87-0.92)	<0.001	0	0.369
Ovarian cancer	3	0.94	(0.73-1.21)	0.61	75.5	0.017
Endometrial cancer	1	0.87	(0.63-1.20)	0.391	-	-
Liver cancer	3	0.93	(0.50-1.71)	0.808	57.3	0.096
Nuts type						
Peanut	13	0.94	(0.84-1.04)	0.225	67.4	<0.001
Tree nut	8	0.88	(0.79-0.99)	0.03	57.5	0.021
Peanut butter	7	1.06	(0.99-1.13)	0.081	0	0.499

Abbreviations: RR, relative risk; CI, confidence interval.

A significant inverse association was observed between nut intake and cancer risk in women (RR = 0.87; 95% CI, 0.79–0.95; *P* = 0.001), but the protective effect of nuts was not statistically significant in men (RR = 0.92; 95% CI, 0.84–1.01; *P* = 0.080) (Supplementary Figure 6).

On the basis of socioeconomic status, an inverse relationship was observed mainly in developed countries (RR = 0.90; 95% CI, 0.86–0.94; *P* < 0.001), and no significant association was observed in developing countries (RR = 0.90; 95% CI, 0.72–1.13; *P* = 0.365). We also analyzed the effect of nut type on cancer risk, and the results suggested that tree nut consumption was associated with a reduced cancer risk (8 studies, RR = 0.88; 95% CI, 0.79–0.99; *P* = 0.03; I^2^ = 57.5%). However, no significant association was identified in the case of studies on peanut (13 studies, RR = 0.94; 95% CI, 0.84–1.04; *P* = 0.225; I^2^ = 67.4%) and peanut butter (7 studies, RR = 1.06; 95% CI, 0.99–1.13; *P* = 0.081; I^2^ = 0) consumption.

**Figure 3:**
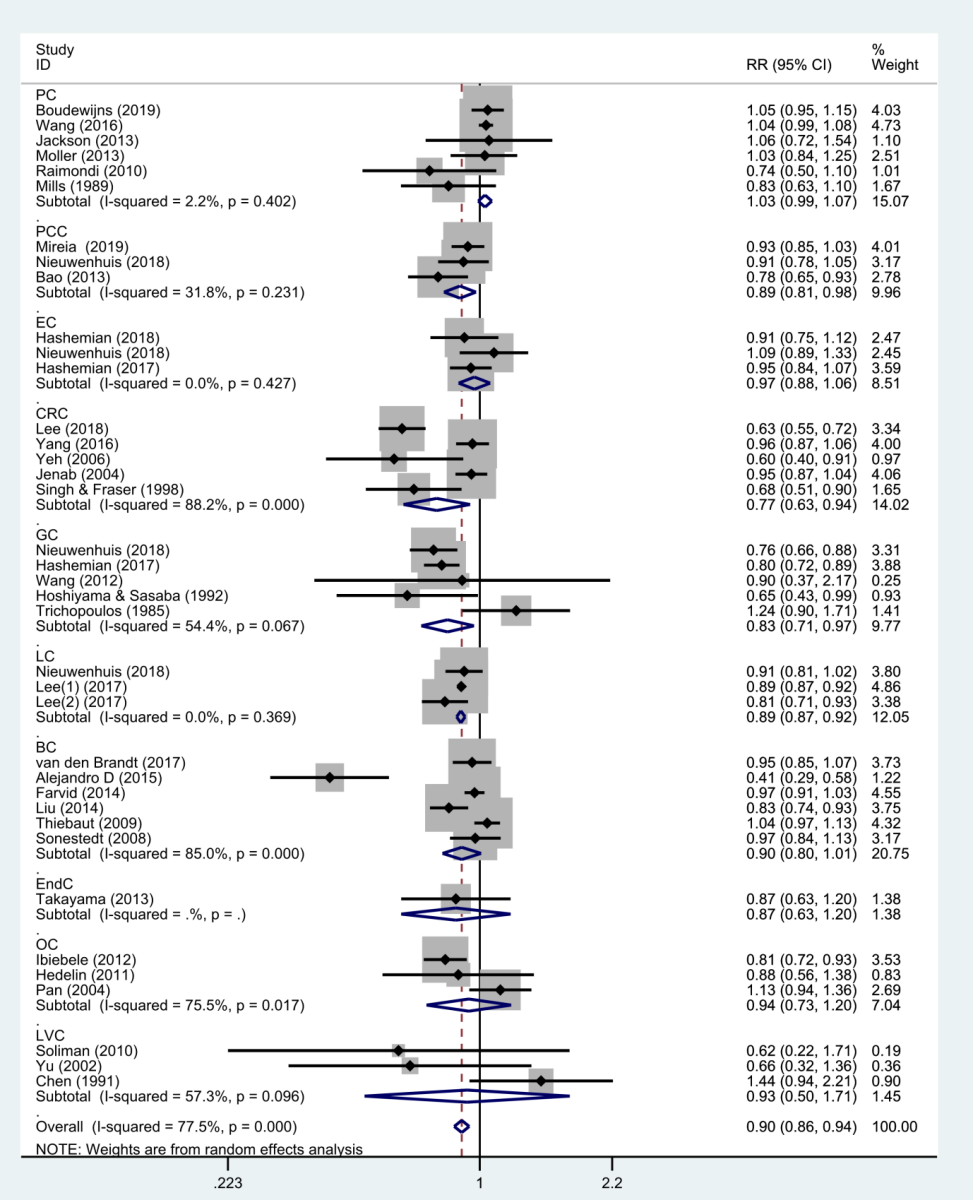
**Subgroup analyses of the association between nut intake and specific types of cancer.** Note: Weights are from the random-effects analysis. Abbreviations: PC, prostate cancer; EC, esophagus cancer; CRC, colorectal cancer; GC, gastric cancer; PCC, pancreatic cancer; LC, lung cancer; BC, breast cancer; EndC, endometrial cancer; OC, ovarian cancer; LVC, liver cancer, RR, relative risk; CI, confidence interval.

Figure 3 shows the results of subgroup analysis stratified by cancer type. Nut consumption was negatively correlated with gastric cancer (5 studies, RR = 0.83; 95% CI, 0.71–0.97; *P* = 0.017), colorectal cancer (5 studies, RR = 0.77; 95% CI, 0.63–0.94; *P* = 0.011), pancreatic cancer (3 studies, RR = 0.89; 95% CI, 0.81–0.98; *P* = 0.015), and lung cancer (3 studies, RR = 0.89; 95% CI, 0.87–0.92; *P* < 0.001). On the contrary, there was no significant association between nut consumption and breast cancer (6 studies, RR = 0.90; 95% CI, 0.80–1.01; *P* = 0.067), esophageal cancer (3 studies, RR = 0.97; 95% CI, 0.88–1.06; *P* = 0.498), prostate cancer (6 studies, RR = 1.03; 95% CI, 0.99–1.07; *P* = 0.139), ovarian cancer (3 studies, RR = 0.94; 95% CI, 0.73–1.21; *P* = 0.610), endometrial cancer (1 study, RR = 0.87; 95% CI, 0.63–1.20; *P* = 0.391), and liver cancer (3 studies, RR = 0.93; 95% CI, 0.50–1.71; *P* = 0.808). Moreover, there was no publication bias in this subgroup analysis (*P* = 0.185 for Egger’s test and *P* = 0.296 for Begg’s test) (Supplementary Figure 7).

### Dose-response analysis

**Figure 4:**
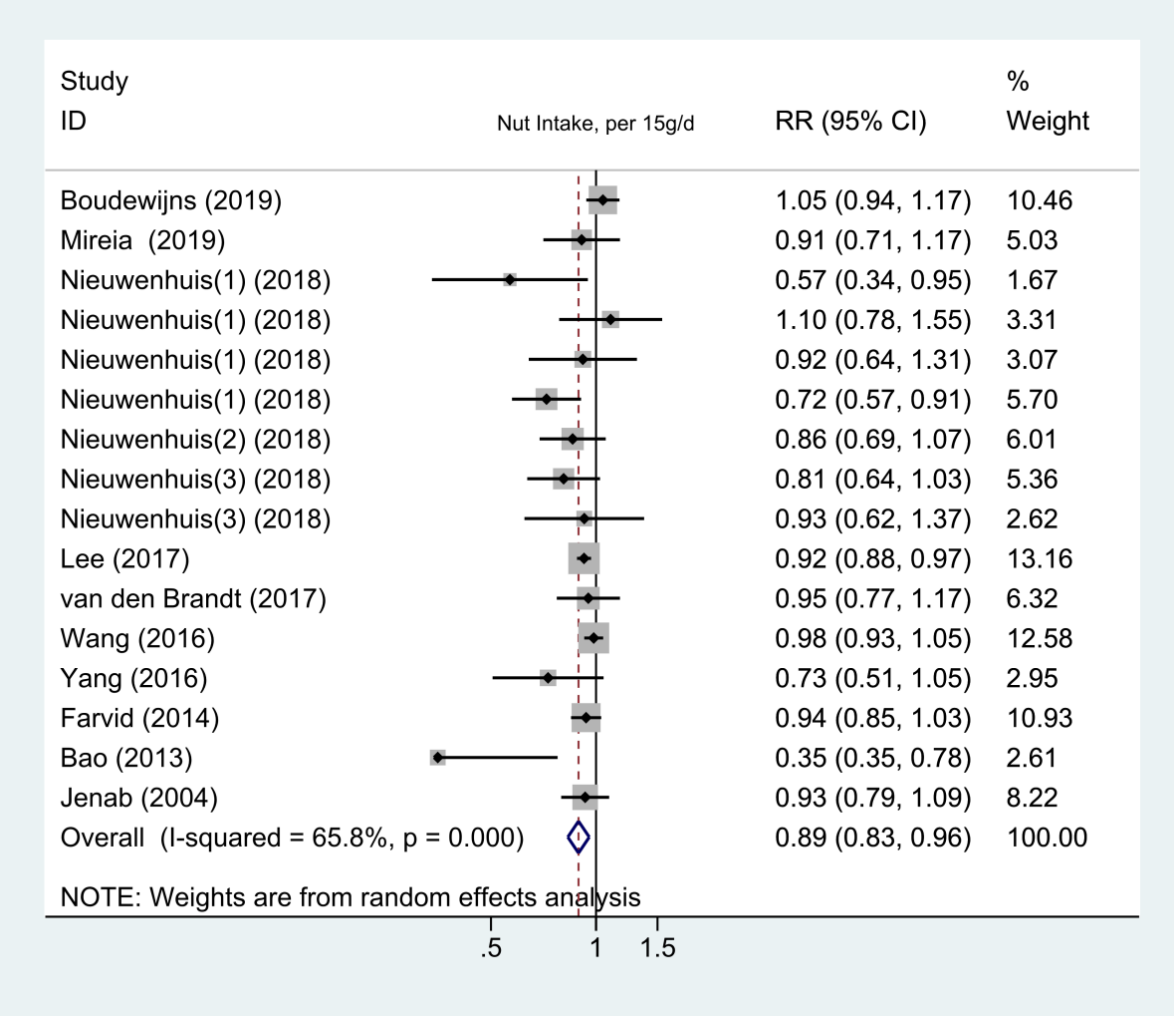
**The meta-analysis of the association between nut intake (per 15 g/day) and risk of cancer.** Note: Weights are from the random-effects analysis. Abbreviations: RR, relative risk; CI, confidence interval.

We conducted an additional dose-response analysis by pooling 16 cohort studies (12 publications, 1 consisting of 4 sub-studies, and 1 publication reporting 2 sub-studies) to confirm the beneficial effect of nuts. We observed interstudy heterogeneity (I^2^ = 77.0%) (Supplementary Figure 8). The findings showed that increasing the intake of nuts by 15 g per day can reduce the risk of cancer by 11% (RR = 0.89; 95% CI, 0.83–0.96; *P* = 0.005) (Figure 4), with little heterogeneity observed. A nonlinear dose-response meta-analysis showed that when nut consumption increased from 0 to 5 g/day (*P*-nonlinearity = 0.0414), the dose-response curve showed an obvious downward trend (Supplementary Figure 9), and further reductions were observed when the intake increased to more than 10 g/d. Because the *P*-value for nonlinearity is critical, we conducted a linear dose-response meta-analysis (Supplementary Figure 10), which also showed a lower trend for risk with higher nut consumption.

### Nut consumption and cancer-specific mortality

**Figure 5:**
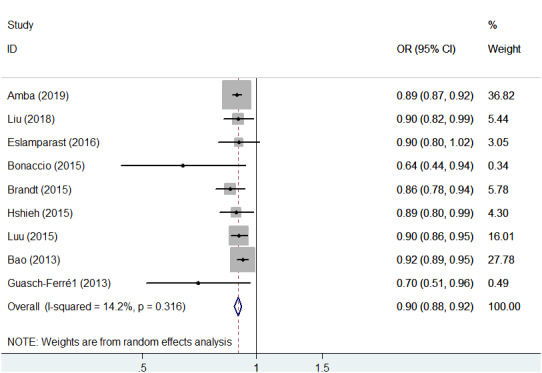
**Association between nut intake and cancer-specific mortality.** Note: Weights are from the random-effects analysis. Abbreviations: OR, odds ratio; CI, confidence interval.

The relevant RRs of nut consumption and cancer mortality were calculated using the random-effects model (Figure 5). The association between nut consumption and cancer-specific mortality was evaluated based on 9 studies, which accounted for 49,161 cancer deaths. There was a significant reduction in the risk of cancer-specific mortality in cases of nut consumption versus no nut consumption (OR = 0.90; 95% CI, 0.88–0.92; *P* < 0.001), and little evidence of heterogeneity (*P* = 0.316, I^2^ = 14.2%) was found in this analysis.

## DISCUSSION

The association between frequent nut consumption and a reduced risk of chronic diseases such as cardiovascular disease [8] and type 2 diabetes [8] has been well established. Nut intake does not cause weight gain, but it improves body mass index and reduces the risk of obesity [6, 17]. There has been a recent public increase in interest in the association between nut consumption and cancer [8, 18–23]. Recent studies have indicated a significant association between nut consumption and cancer risk and mortality [4, 8]. A meta-analysis including 36 observational studies in 2015 on nut consumption, cancer risk, and type 2 diabetes mellitus showed that nut intake can reduce the risk of colorectal, endometrial, and pancreatic cancers [4]. Thirteen studies have been published since 2015, of which 9 reported no significant association with cancer types. Further studies are required to determine the association between different nut categories and specific cancer types. Moreover, unlike previous meta-analyses on nut intake and cancer that involved studies on cardiovascular diseases, diabetes, and other metabolic diseases, this meta-analysis, which focused exclusively on the association between nut consumption and the risks of cancer incidence and mortality, is indispensable. Therefore, a comprehensive understanding of this association was attained.

This meta-analysis comprised the most up-to-date and comprehensive studies (47 studies) to assess the association between nut consumption and risk of cancer incidence and cause-specific mortality. Large studies such as the European Prospective Investigation into Cancer and Nutrition (EPIC) [24], PREvención con DIeta MEDiterránea study (PREDIMED clinical trial) [25], Nurses’ Health Study, and Health Professionals Follow-up Study (the NHS and the HPFS) [26] were included in the analysis. Our meta-analysis indicated that frequent nut consumption was significantly associated with a reduced total risk of cancer. Compared to 9 cohort studies (18,490 cases and 304,285 participants) in a previous meta-analysis [8], we included 16 cohort studies (43,328 cases and 1,903,651 participants) in our dose-response analysis of cancer risk and nut intake. We found that there was a significant association between increased nut intake and reduced cancer risk. Furthermore, the dose-response relationship was nonlinear. This finding is similar to that of a previous study [8] showing nonlinear associations between nut intake and cardiovascular disease risk, stroke risk, and all-cause mortality.

A large number of recent studies have described the anticancer mechanisms of nuts, stating that nuts inhibit cancer progression by altering lipid metabolism. Other recent studies have reported that nuts reduce the ingestion of low-density lipoprotein (LDL) and elementary fatty acids [6], thereby lowering cholesterol levels, reducing the storage of cholesteryl ethers in cancer cells, and possibly inhibiting tumor cell proliferation and cancer development. Moreover, studies have shown that a high intake of nuts can reduce endothelial dysfunction [27], lipid peroxidation [28], and insulin resistance [29]. Some studies recently found that oxidative damage and insulin resistance facilitate the occurrence and development of cancers [30]. The antioxidant components [31] present in nuts have moderate antioxidant capacity and reduce oxidative DNA damage [6] and cell proliferation [32] while decreasing inflammatory reactions [33] and the accumulation of circulatory insulin-like growth factor 1 [34]. Furthermore, the anticancer antioxidant components in nuts can induce apoptosis [35], inhibit angiogenesis [36], and change the gut microbiota [37]. This evidence supports the claim that nuts play an important role in cancer prevention.

Due to variability in study design, we conducted a subgroup analysis by classifying the studies into cohort and case-control categories. No statistically significant difference was observed among the subgroups. Considering the variations in public health, medical status, and quality of life among different countries, subgroup analyses were conducted according to socioeconomic status. An inverse association between cancer and nut intake was observed in developed countries but not in developing countries. Since a low socioeconomic status leads to low health care utilization and quality of life, a decreased focus on health, and low nut consumption due to the high cost of beneficial tree nuts in the universal market, socioeconomic inequality leads to differences in cancer incidence [2] and nut consumption levels. Hence, improving the distribution of medical services and eradicating the socioeconomic disparities among disadvantaged groups and countries would undoubtedly accelerate the progress against cancer.

A significant inverse association between nut intake and cancer risk was observed in women but not in men, with no evidence of statistically significant heterogeneity. Similarly, the NHS, a large prospective study with 83,818 female participants, indicated that nut intake may reduce the risk of diabetes mellitus in women by 29% [9]. However, the Physicians’ Health Study, a prospective cohort study of 20,224 male subjects, reported no significant association between nut consumption and the incidence of diabetes mellitus [38]. Moreover, the results from the BMES, a population-based cohort study with subgroup analyses of 2,893 participants, reported a statistically significant association between nut consumption and specific mortality from cardiovascular disease (CVD) and stroke in women but not in men [39]. The sex-specific discrepancy observed in the studies on diabetes mellitus, cancer, CVD, and stroke has not been fully explained in these studies [9, 38, 39]. It is speculated that the diverse reproductive hormone milieu may account for the observed sex-based discrepancy [39]. Moreover, the contingency may contribute to sex-based differences; for example, different sample sizes between men and women. Furthermore, the possibility of developing several types of cancers including gastric, colorectal, pancreatic, and lung cancers is higher in men than in women, which may account for this difference. Further studies on these sex-based differences are required.

We also conducted a subgroup analysis of the specific types of cancer. Inconsistent with a similar meta-analysis in 2015 [4], inverse associations were observed between nut intake and the risk of gastric, lung, colorectal, and pancreatic cancers. A statistically significant association was found between nut intake and a lower risk of endometrial cancer after pooling of the estimates from two included studies on endometrial cancer. Our study included only one study on endometrial cancer; another study was excluded because it examined nut intake combined with the intake of other foods. Therefore, given the limited evidence, we did not find an association indicating a reduced risk of endometrial cancer. We identified a significant inverse association between a reduced cancer risk and the consumption of tree nuts but not with the consumption of peanuts or peanut butter. Among the included studies, there were more studies on peanuts than on tree nuts; thus, this result may not be statistically significant. A randomized-controlled trial [40] suggested that different types of nuts exert different effects on cardiovascular risk and that only tree nuts were related to the reduced risk of cancer, which supports our results to a certain extent. Because of the limited evidence on specific nut subtypes, further research studies on specific nuts and their association with different cancer types and other health outcomes are warranted.

We detected inter-study heterogeneity in this meta-analysis, probably because of several confounding factors. Subgroup analyses were conducted to explore the sources of heterogeneity among the included studies stratified by study design, sex, socioeconomic status, cancer type, and nut category. And we also regressed the study characteristics including study quality scores and adjustment for age. The sensitivity analyses were also performed for all studies and the studies published before and after 2010. Considering there were no qualitative changes in people's lifestyles and eating habits before and after 2010, and the two groups results showed largely coincident confidence intervals, so the findings cannot show a statistically significant difference before and after 2010. And it may have been a false negative results of the older studies. What’s more, no significant source of heterogeneity was identified. Therefore, we calculated the summary RRs with 95% CIs using the random-effects model to reduce deviations in the association. Moreover, we found no significant publication bias. The consistent results and sensitivity obtained from several subgroup analyses confirm the robustness and reliability of our study.

The main safety considerations of an increased nut intake are possible weight gain, anaphylactic reaction, and potential toxicity [41], particularly by aflatoxins. Contrary to expectations, studies have found a significant relationship between frequent nut consumption and reduced weight gain [7]. Moreover, studies have identified allergenic seed storage proteins that elicit the production of specific immunoglobulin E antibodies [42], which are responsible for allergic reactions to nuts. Once nut allergy is firmly established, prevention of subsequent episodes tends to be clinically worse, especially for children. Therefore, increasing one’s nut intake should only be recommended for affected patients when an anaphylactic reaction is absent. Aflatoxin [43] contamination must be avoided as well. The Dietary Guidelines for Americans recommend a minimum portion of 30 g/day of nuts, seeds, and legumes since they may have beneficial effects on human health [44]. However, in the current studies, only 2% of nut consumers reported an intake >30 g/day. Moreover, as per the dietary recommendation of the American Heart Association [45], our study recommends the consumption of 4–5 servings of nuts (1 serving = 28 g) every week as part of a healthy diet.

Several studies have assessed the relationship between nut consumption and cancer-specific mortality, albeit with discordant results. The findings of the BMES, in which participants were followed up for 15 years, indicated no significant correlation between nut consumption and cancer-specific mortality [39]. However, after performing both fixed-effects and random-effects in our meta-analysis, the similar results show that nut intake exerted a beneficial effect on reducing cancer mortality, which is really a sign of robustness and show no statistically significant heterogeneity existing. Additionally, the results from the NHS and HPFS [26] were partly in line with our results. In short, our results confirm that nut intake can decrease cancer-specific mortality and suggest increasing one’s nut intake to promote health.

The strengths of this study are as follows. First, compared with previous meta-analyses including other diseases, this is the first meta-analysis to exclusively assess the correlation between nut consumption and cancer occurrence and mortality. Second, the included studies are of high quality and have large sample sizes, which enables an effective assessment. Third, the pooled RRs with 95% CIs were calculated for comparisons between nut consumption and non-consumption instead of those between the highest and lowest categories of nut consumption, which can exaggerate the correlations. Fourth, we updated the dose-response analysis of nut intake and cancer risk. In addition, we had sufficient statistical data to detect significant correlations in diverse subgroups stratified by study design, sex, socioeconomic status, cancer type, and nut category.

This study has potential limitations that warrant consideration. First, we did not search for unpublished studies or unconventional articles, which are difficult to obtain. Hence, a relatively conservative conclusion was drawn on the basis of the available data. Second, nut intake information was not updated in a timely manner during the course of follow-up in some of the cohort studies included in the analysis, and recall bias may exist in the case-control studies, which may result in deviations of estimates from actual nut consumption. Third, despite subgroup and sensitivity analyses, the source of heterogeneity remains unknown, although it might be explained by other factors (such as differences in races, BMI values, measurement of nut intake in individual studies, consumed nut quality). Fourth, since only one study reported the relevant RRs for endometrial cancer, we could not obtain combined results for the risk of endometrial cancer. Finally, studies with negative results may not have been published, resulting in potential publication bias.

In conclusion, the long-term consumption of nuts appears to be significantly correlated with a reduced risk of cancer incidence and mortality, as higher intake of nuts was significantly associated with a lower cancer risk. Furthermore, increasing one’s intake of nuts by 15 g per day may greatly benefit human health.

## MATERIALS AND METHODS

This study was conducted according to the guidelines provided by the Preferred Reporting Items for Systematic Review and Meta-Analysis statement [46]. Two authors (Dai Zhang and Cong Dai) independently performed the literature search, study selection, data extraction, and quality assessment. Any disagreements were resolved by consulting a senior investigator (Zhijun Dai). Ethical approval was not required for this meta-analysis.

### Literature search

We searched PubMed, the Web of Science, and Embase databases for relevant studies on nut consumption and cancer published up to August 2019. The terms “cancer(s)” [Title/Abstract], “tumor(s)” [Title/Abstract], and “neoplasm(s)” [Title/Abstract] in combination with “Nuts” [Mesh], “nut(s)” [Title/Abstract], “peanut(s)” [Title/Abstract], “tree nut(s)” [Title/Abstract], “Walnut(s)” [Title/Abstract], “pecan(s)” [Title/Abstract], “almond(s)” [Title/Abstract], “pistachio(s)” [Title/Abstract], “coconut(s)” [Title/Abstract], “macadamia nuts” [Title/Abstract], and “cashew(s)” [Title/Abstract] were used in the search. No restrictions were imposed except English language publication and inclusion of human subjects. We manually reviewed the reference lists of all relevant articles to ensure that no relevant studies were missed.

### Eligibility criteria

Studies that met the following criteria were included in the meta-analysis: (1) observational or clinical design; (2) nut intake as the exposure factor and cancer (pathological diagnosis) as the outcome; and (3) availability of relevant risk estimates (relative risks [RRs]/odds ratio [ORs]/hazard ratios [HRs] and 95% confidence intervals [CIs]) of cancer incidence or mortality in relation to nut consumption. The exclusion criteria were as follows: (1) incomplete or unclear RR estimates with insufficient information to calculate them; (2) nut consumption assessment performed in combination with that for other food groups; and (3) cross-sectional studies, animal studies, systematic reviews, meta-analyses, letters, and commentaries. When more than one study was conducted on the same cohort, we included the study with the most complete data or the longest follow-up duration.

### Data extraction

Relevant information including author names, year of publication, study country, study design, adjusted ORs/RRs/HRs, and 95% CIs were extracted independently by two investigators (Dai Zhang and Cong Dai). Data on characteristics of the study population including sample size, age, duration of follow-up, nut intake levels, nut types, specific cancer types, and adjusted confounding factors were extracted. If a study provided several risk estimates, we used the estimate from the major multivariable model, which included a greater number of adjusted confounders. Disagreements were resolved by consulting a senior researcher.

### Quality assessment

The qualities of the cohort and case-control studies included in the meta-analysis were assessed by the Newcastle-Ottawa Scale [47]. The scores ranged from 0 to 9 and included study population selection (4 points), inter-group comparability (2 points), and outcome measurements (3 points). Scores of 0–3, 4–6, and 7–9 points indicated low-, medium-, and high-quality studies, respectively.

### Statistical analyses

RRs and ORs with 95% CIs for all nut intake categories were calculated. The average of the natural logarithm of the RRs was estimated, and the RR from each study was weighted by the inverse of its variance. The pooled estimates with 95% CIs for nut intake versus no intake were calculated using fixed-effects and random-effects models [48]. Consumers were defined as those who reported a mean intake > 0 g/day. Total nut intake was analyzed as a continuous variable (15 g/day; 15-g increments correspond to half a standard serving) [49] and as a categorical variable with all non-consumers as the reference category, according to the distribution of total nut intake in most studies. Furthermore, the risk estimates of different nut intakes in each study were pooled and subjected to the final analysis. Heterogeneity across the included studies was assessed using the Q and I^2^ statistics [50]. Heterogeneity was considered significant at *P* values < 0.05 in the Q statistic or I^2^ values ≥ 50%. Subsequently, the random-effects model was selected to calculate the pooled estimates and 95% CIs. A fixed-effects model was selected when no significant heterogeneity was observed and/or the question when it was justified by the clinical questions [51, 52]. To explore the potential origins of heterogeneity, subgroup analyses were conducted with classifications based on study design, cancer type, nut type, sex, and socioeconomic status of the study location; the meta-regression analyses were performed according to some study characteristics including study quality scores and adjustment for age.

For the linear dose-response meta-analysis, we used the generalized least squares for trend method [53]. The original studies were required to have at least three intake groups with the scope for extracting five types of variable data, including the dose group of nut intake, number of cases in each group, corresponding person-years, RRs, and 95% CIs. To unify the unit (g/d), we converted the dosage unit by the standard 28 g/serving according to 4 included studies [10–56], the units in which were not g/d. A dose value for nut consumption in each category was assigned as suggested by Cao et al. [57]. When nut consumption was reported in closed intervals, the midpoint between the upper and lower lines of the interval is considered the average exposure level. For the upper open interval, the midpoint of the category was set at 1.5-times the lower bound value. For the lower open interval, the midpoint of the category was set at 1.5-times the upper bound value [58]. A nonlinear dose-response meta-analysis was performed using a restricted cubic spline regression model. With the consideration of different segmentation points for independent variables, these variables were divided into different small segments and fitted to regression models [59]. Multivariate dose-response meta-analyses were performed using linear regression and nonlinear regression models to match the data.

A sensitivity analysis was performed to assess the stability of the results and identify the latent sources of heterogeneity by excluding the included individual studies one by one. Publication bias was detected using Egger’s linear regression test or Begg’s test [60] and funnel plots. A two-sided significance level of <0.05 was used for all tests. All meta-analyses were performed using STATA statistical software (version 14.0; StataCorp, College Station, TX, USA). The original data for this meta-analysis are shown in Supplementary Tables 1, 2.

## Supplementary Material

Supplementary Figures

Supplementary Table 1

Supplementary Table 2

## References

[1] Bray F, Ferlay J, Soerjomataram I, Siegel RL, Torre LA, Jemal A. Global cancer statistics 2018: GLOBOCAN estimates of incidence and mortality worldwide for 36 cancers in 185 countries. CA Cancer J Clin. 2018; 68:394–424. 10.3322/caac.21492 30207593

[2] Siegel RL, Miller KD, Jemal A. Cancer statistics, 2019. CA Cancer J Clin. 2019; 69:7–34. 10.3322/caac.21551 30620402

[3] de Souza RG, Schincaglia RM, Pimentel GD, Mota JF. Nuts and human health outcomes: a systematic review. Nutrients. 2017; 9:1311. 10.3390/nu9121311 29207471 PMC5748761

[4] Wu L, Wang Z, Zhu J, Murad AL, Prokop LJ, Murad MH. Nut consumption and risk of cancer and type 2 diabetes: a systematic review and meta-analysis. Nutr Rev. 2015; 73:409–25. 10.1093/nutrit/nuv006 26081452 PMC4560032

[5] Ros E. Health benefits of nut consumption. Nutrients. 2010; 2:652–82. 10.3390/nu2070652 22254047 PMC3257681

[6] Falasca M, Casari I, Maffucci T. Cancer chemoprevention with nuts. J Natl Cancer Inst. 2014; 106:dju238. 10.1093/jnci/dju238 25210199

[7] Bes-Rastrollo M, Sabaté J, Gómez-Gracia E, Alonso A, Martínez JA, Martínez-González MA. Nut consumption and weight gain in a mediterranean cohort: the SUN study. Obesity (Silver Spring). 2007; 15:107–16. 10.1038/oby.2007.507 17228038

[8] Aune D, Keum N, Giovannucci E, Fadnes LT, Boffetta P, Greenwood DC, Tonstad S, Vatten LJ, Riboli E, Norat T. Nut consumption and risk of cardiovascular disease, total cancer, all-cause and cause-specific mortality: a systematic review and dose-response meta-analysis of prospective studies. BMC Med. 2016; 14:207. 10.1186/s12916-016-0730-3 27916000 PMC5137221

[9] Jiang R, Manson JE, Stampfer MJ, Liu S, Willett WC, Hu FB. Nut and peanut butter consumption and risk of type 2 diabetes in women. JAMA. 2002; 288:2554–60. 10.1001/jama.288.20.2554 12444862

[10] Lee JT, Lai GY, Liao LM, Subar AF, Bertazzi PA, Pesatori AC, Freedman ND, Landi MT, Lam TK. Nut consumption and lung cancer risk: results from two large observational studies. Cancer Epidemiol Biomarkers Prev. 2017; 26:826–36. 10.1158/1055-9965.EPI-16-0806 28077426 PMC6020049

[11] Hedelin M, Löf M, Andersson TM, Adlercreutz H, Weiderpass E. Dietary phytoestrogens and the risk of ovarian cancer in the women's lifestyle and health cohort study. Cancer Epidemiol Biomarkers Prev. 2011; 20:308–17. 10.1158/1055-9965.EPI-10-0752 21098648

[12] Boudewijns EA, Nieuwenhuis L, Geybels MS, van den Brandt PA. Total nut, tree nut, peanut, and peanut butter intake and the risk of prostate cancer in the Netherlands cohort study. Prostate Cancer Prostatic Dis. 2019; 22:467–74. 10.1038/s41391-019-0131-8 30692586

[13] Chen GC, Zhang R, Martínez-González MA, Zhang ZL, Bonaccio M, van Dam RM, Qin LQ. Nut consumption in relation to all-cause and cause-specific mortality: a meta-analysis 18 prospective studies. Food Funct. 2017; 8:3893–905. 10.1039/c7fo00915a 28875220

[14] Grosso G, Yang J, Marventano S, Micek A, Galvano F, Kales SN. Nut consumption on all-cause, cardiovascular, and cancer mortality risk: a systematic review and meta-analysis of epidemiologic studies. Am J Clin Nutr. 2015; 101:783–93. 10.3945/ajcn.114.099515 25833976

[15] Davis PA, Jenab M, Vanden Heuvel JP, Furlong T, Taylor S. Tree nut and peanut consumption in relation to chronic and metabolic diseases including allergy. J Nutr. 2008; 138:1757S–62S. 10.1093/jn/138.9.1757S 18716182

[16] http://www.uwm.edu.pl/edu/lidiawadolowska/.

[17] Casas-Agustench P, Bulló M, Ros E, Basora J, Salas-Salvadó J, and Nureta-PREDIMED investigators. Cross-sectional association of nut intake with adiposity in a mediterranean population. Nutr Metab Cardiovasc Dis. 2011; 21:518–25. 10.1016/j.numecd.2009.11.010 20219336

[18] Hoshiyama Y, Sasaba T. A case-control study of stomach cancer and its relation to diet, cigarettes, and alcohol consumption in saitama prefecture, Japan. Cancer Causes Control. 1992; 3:441–48. 10.1007/BF00051357 1525325

[19] Bonaccio M, Di Castelnuovo A, De Curtis A, Costanzo S, Bracone F, Persichillo M, Donati MB, de Gaetano G, Iacoviello L, and Moli-sani Project investigators. Nut consumption is inversely associated with both cancer and total mortality in a mediterranean population: prospective results from the moli-sani study. Br J Nutr. 2015; 114:804–11. 10.1017/S0007114515002378 26313936

[20] Nieuwenhuis L, van den Brandt PA. Nut and peanut butter consumption and the risk of lung cancer and its subtypes: a prospective cohort study. Lung Cancer. 2019; 128:57–66. 10.1016/j.lungcan.2018.12.018 30642454

[21] Nieuwenhuis L, van den Brandt PA. Total nut, tree nut, peanut, and peanut butter consumption and the risk of pancreatic cancer in the Netherlands cohort study. Cancer Epidemiol Biomarkers Prev. 2018; 27:274–84. 10.1158/1055-9965.EPI-17-0448 29358224

[22] Liu G, Guasch-Ferré M, Hu Y, Li Y, Hu FB, Rimm EB, Manson JE, Rexrode KM, Sun Q. Nut consumption in relation to cardiovascular disease incidence and mortality among patients with diabetes mellitus. Circ Res. 2019; 124:920–29. 10.1161/CIRCRESAHA.118.314316 30776978 PMC6417933

[23] Hashemian M, Murphy G, Etemadi A, Poustchi H, Sharafkhah M, Kamangar F, Pourshams A, Malekshah AF, Khoshnia M, Gharavi A, Hekmatdoost A, Brennan PJ, Boffetta P, et al. Nut consumption and the risk of oesophageal squamous cell carcinoma in the golestan cohort study. Br J Cancer. 2018; 119:176–81. 10.1038/s41416-018-0148-0 29950612 PMC6048068

[24] Jenab M, Ferrari P, Slimani N, Norat T, Casagrande C, Overad K, Olsen A, Stripp C, Tjønneland A, Boutron-Ruault MC, Clavel-Chapelon F, Kesse E, Nieters A, et al. Association of nut and seed intake with colorectal cancer risk in the european prospective investigation into cancer and nutrition. Cancer Epidemiol Biomarkers Prev. 2004; 13:1595–603. 15466975

[25] Guasch-Ferré M, Bulló M, Martínez-González MÁ, Ros E, Corella D, Estruch R, Fitó M, Arós F, Wärnberg J, Fiol M, Lapetra J, Vinyoles E, Lamuela-Raventós RM, et al, and PREDIMED study group. Frequency of nut consumption and mortality risk in the PREDIMED nutrition intervention trial. BMC Med. 2013; 11:164. 10.1186/1741-7015-11-164 23866098 PMC3738153

[26] Bao Y, Han J, Hu FB, Giovannucci EL, Stampfer MJ, Willett WC, Fuchs CS. Association of nut consumption with total and cause-specific mortality. N Engl J Med. 2013; 369:2001–11. 10.1056/NEJMoa1307352 24256379 PMC3931001

[27] Jenkins DJ, Kendall CW, Marchie A, Josse AR, Nguyen TH, Faulkner DA, Lapsley KG, Blumberg J. Almonds reduce biomarkers of lipid peroxidation in older hyperlipidemic subjects. J Nutr. 2008; 138:908–13. 10.1093/jn/138.5.908 18424600

[28] Jenkins DJ, Kendall CW, Josse AR, Salvatore S, Brighenti F, Augustin LS, Ellis PR, Vidgen E, Rao AV. Almonds decrease postprandial glycemia, insulinemia, and oxidative damage in healthy individuals. J Nutr. 2006; 136:2987–92. 10.1093/jn/136.12.2987 17116708

[29] Rajaram S, Sabaté J. Nuts, body weight and insulin resistance. Br J Nutr. 2006 (Suppl 2); 96:S79–86. 10.1017/bjn20061867 17125537

[30] Calle EE, Kaaks R. Overweight, obesity and cancer: epidemiological evidence and proposed mechanisms. Nat Rev Cancer. 2004; 4:579–91. 10.1038/nrc1408 15286738

[31] Carlsen MH, Halvorsen BL, Holte K, Bøhn SK, Dragland S, Sampson L, Willey C, Senoo H, Umezono Y, Sanada C, Barikmo I, Berhe N, Willett WC, et al. The total antioxidant content of more than 3100 foods, beverages, spices, herbs and supplements used worldwide. Nutr J. 2010; 9:3. 10.1186/1475-2891-9-3 20096093 PMC2841576

[32] Vanden Heuvel JP, Belda BJ, Hannon DB, Kris-Etherton PM, Grieger JA, Zhang J, Thompson JT. Mechanistic examination of walnuts in prevention of breast cancer. Nutr Cancer. 2012; 64:1078–86. 10.1080/01635581.2012.717679 23061909

[33] Colpo E, Dalton D A Vilanova C, Reetz LG, Duarte MM, Farias IL, Meinerz DF, Mariano DO, Vendrusculo RG, Boligon AA, Dalla Corte CL, Wagner R, Athayde ML, da Rocha JB. Brazilian nut consumption by healthy volunteers improves inflammatory parameters. Nutrition. 2014; 30:459–65. 10.1016/j.nut.2013.10.005 24607303

[34] Kim H, Yokoyama W, Davis PA. TRAMP prostate tumor growth is slowed by walnut diets through altered IGF-1 levels, energy pathways, and cholesterol metabolism. J Med Food. 2014; 17:1281–86. 10.1089/jmf.2014.0061 25354213 PMC4259176

[35] Chen HS, Bai MH, Zhang T, Li GD, Liu M. Ellagic acid induces cell cycle arrest and apoptosis through TGF-β/Smad3 signaling pathway in human breast cancer MCF-7 cells. Int J Oncol. 2015; 46:1730–38. 10.3892/ijo.2015.2870 25647396

[36] Nagel JM, Brinkoetter M, Magkos F, Liu X, Chamberland JP, Shah S, Zhou J, Blackburn G, Mantzoros CS. Dietary walnuts inhibit colorectal cancer growth in mice by suppressing angiogenesis. Nutrition. 2012; 28:67–75. 10.1016/j.nut.2011.03.004 21795022 PMC3237820

[37] Ukhanova M, Wang X, Baer DJ, Novotny JA, Fredborg M, Mai V. Effects of almond and pistachio consumption on gut microbiota composition in a randomised cross-over human feeding study. Br J Nutr. 2014; 111:2146–52. 10.1017/S0007114514000385 24642201

[38] Kochar J, Gaziano JM, Djoussé L. Nut consumption and risk of type II diabetes in the physicians’ health study. Eur J Clin Nutr. 2010; 64:75–79. 10.1038/ejcn.2009.121 19756028 PMC2802656

[39] Gopinath B, Flood VM, Burlutksy G, Mitchell P. Consumption of nuts and risk of total and cause-specific mortality over 15 years. Nutr Metab Cardiovasc Dis. 2015; 25:1125–31. 10.1016/j.numecd.2015.09.006 26607701

[40] Sabaté J, Oda K, Ros E. Nut consumption and blood lipid levels: a pooled analysis of 25 intervention trials. Arch Intern Med. 2010; 170:821–27. 10.1001/archinternmed.2010.79 20458092

[41] Molyneux RJ, Mahoney N, Kim JH, Campbell BC. Mycotoxins in edible tree nuts. Int J Food Microbiol. 2007; 119:72–78. 10.1016/j.ijfoodmicro.2007.07.028 17719114

[42] Zuidmeer L, Goldhahn K, Rona RJ, Gislason D, Madsen C, Summers C, Sodergren E, Dahlstrom J, Lindner T, Sigurdardottir ST, McBride D, Keil T. The prevalence of plant food allergies: a systematic review. J Allergy Clin Immunol. 2008; 121:1210–1218.e4. 10.1016/j.jaci.2008.02.019 18378288

[43] Dorner JW. Management and prevention of mycotoxins in peanuts. Food addit contam part A chem anal control expo risk assess. 2008; 25:203–08. 10.1080/02652030701658357 18286410

[44] Mosher AL, Piercy KL, Webber BJ, Goodwin SK, Casavale KO, Olson RD. Dietary guidelines for americans: implications for primary care providers. Am J Lifestyle Med. 2014; 10:23–35. 10.1177/1559827614521755 30202257 PMC6124854

[45] Goff DC Jr, Lloyd-Jones DM, Bennett G, Coady S, D’Agostino RB Sr, Gibbons R, Greenland P, Lackland DT, Levy D, O’Donnell CJ, Robinson JG, Schwartz JS, Shero ST, et al. 2013 ACC/AHA guideline on the assessment of cardiovascular risk: a report of the american college of cardiology/american heart association task force on practice guidelines. J Am Coll Cardiol. 2014; 63:2935–59. 10.1016/j.jacc.2013.11.005 24239921 PMC4700825

[46] Knobloch K, Yoon U, Vogt PM. Preferred reporting items for systematic reviews and meta-analyses (PRISMA) statement and publication bias. J Craniomaxillofac Surg. 2011; 39:91–92. 10.1016/j.jcms.2010.11.001 21145753

[47] Stang A. Critical evaluation of the newcastle-ottawa scale for the assessment of the quality of nonrandomized studies in meta-analyses. Eur J Epidemiol. 2010; 25:603–05. 10.1007/s10654-010-9491-z 20652370

[48] DerSimonian R, Laird N. Meta-analysis in clinical trials. Control Clin Trials. 1986; 7:177–88. 10.1016/0197-2456(86)90046-2 3802833

[49] World Cancer Research Fund/American Institute for Cancer Research. Continuous Update Project Expert Report 2018. Recommendations and public health and policy implications. 2018.

[50] Higgins JP, Thompson SG, Deeks JJ, Altman DG. Measuring inconsistency in meta-analyses. BMJ. 2003; 327:557–60. 10.1136/bmj.327.7414.557 12958120 PMC192859

[51] von Hippel PT. The heterogeneity statistic I(2) can be biased in small meta-analyses. BMC Med Res Methodol. 2015; 15:35. 10.1186/s12874-015-0024-z 25880989 PMC4410499

[52] Kenneth R, Higgins JP, Thomas L. A re-evaluation of fixed effect(s) meta-analysis. J R Statist Soc A. 2017 181:205–27. 10.1111/rssa.12275

[53] Greenland S, Longnecker MP. Methods for trend estimation from summarized dose-response data, with applications to meta-analysis. Am J Epidemiol. 1992; 135:1301–09. 10.1093/oxfordjournals.aje.a116237 1626547

[54] Wang W, Yang M, Kenfield SA, Hu FB, Stampfer MJ, Willett WC, Fuchs CS, Giovannucci EL, Bao Y. Nut consumption and prostate cancer risk and mortality. Br J Cancer. 2016; 115:371–74. 10.1038/bjc.2016.181 27280637 PMC4973153

[55] Yang M, Hu FB, Giovannucci EL, Stampfer MJ, Willett WC, Fuchs CS, Wu K, Bao Y. Nut consumption and risk of colorectal cancer in women. Eur J Clin Nutr. 2016; 70:333–37. 10.1038/ejcn.2015.66 25944181 PMC4892359

[56] Bao Y, Hu FB, Giovannucci EL, Wolpin BM, Stampfer MJ, Willett WC, Fuchs CS. Nut consumption and risk of pancreatic cancer in women. Br J Cancer. 2013; 109:2911–16. 10.1038/bjc.2013.665 24149179 PMC3844914

[57] Cao S, Liu L, Yin X, Wang Y, Liu J, Lu Z. Coffee consumption and risk of prostate cancer: a meta-analysis of prospective cohort studies. Carcinogenesis. 2014; 35:256–61. 10.1093/carcin/bgt482 24343360

[58] Liu H, Hu GH, Wang XC, Huang TB, Xu L, Lai P, Guo ZF, Xu YF. Coffee consumption and prostate cancer risk: a meta-analysis of cohort studies. Nutr Cancer. 2015; 67:392–400. 10.1080/01635581.2015.1004727 25706900

[59] Jackson D, White IR, Thompson SG. Extending DerSimonian and laird’s methodology to perform multivariate random effects meta-analyses. Stat Med. 2010; 29:1282–97. 10.1002/sim.3602 19408255

[60] Egger M, Davey Smith G, Schneider M, Minder C. Bias in meta-analysis detected by a simple, graphical test. BMJ. 1997; 315:629–34. 10.1136/bmj.315.7109.629 9310563 PMC2127453

