# Supplementary material for: Meta-analysis of the association between nut consumption and the risks of cancer incidence and cancer-specific mortality
Source: Aging (Albany NY). 2020 Jun 2;12(11):10772–94. doi: 10.18632/aging.103292 (PMC7346045; doi:10.18632/aging.103292)
Supplement: Supplementary Figures [file aging-12-103292-s003..pdf]

SUPPLEMENTARY FIGURES

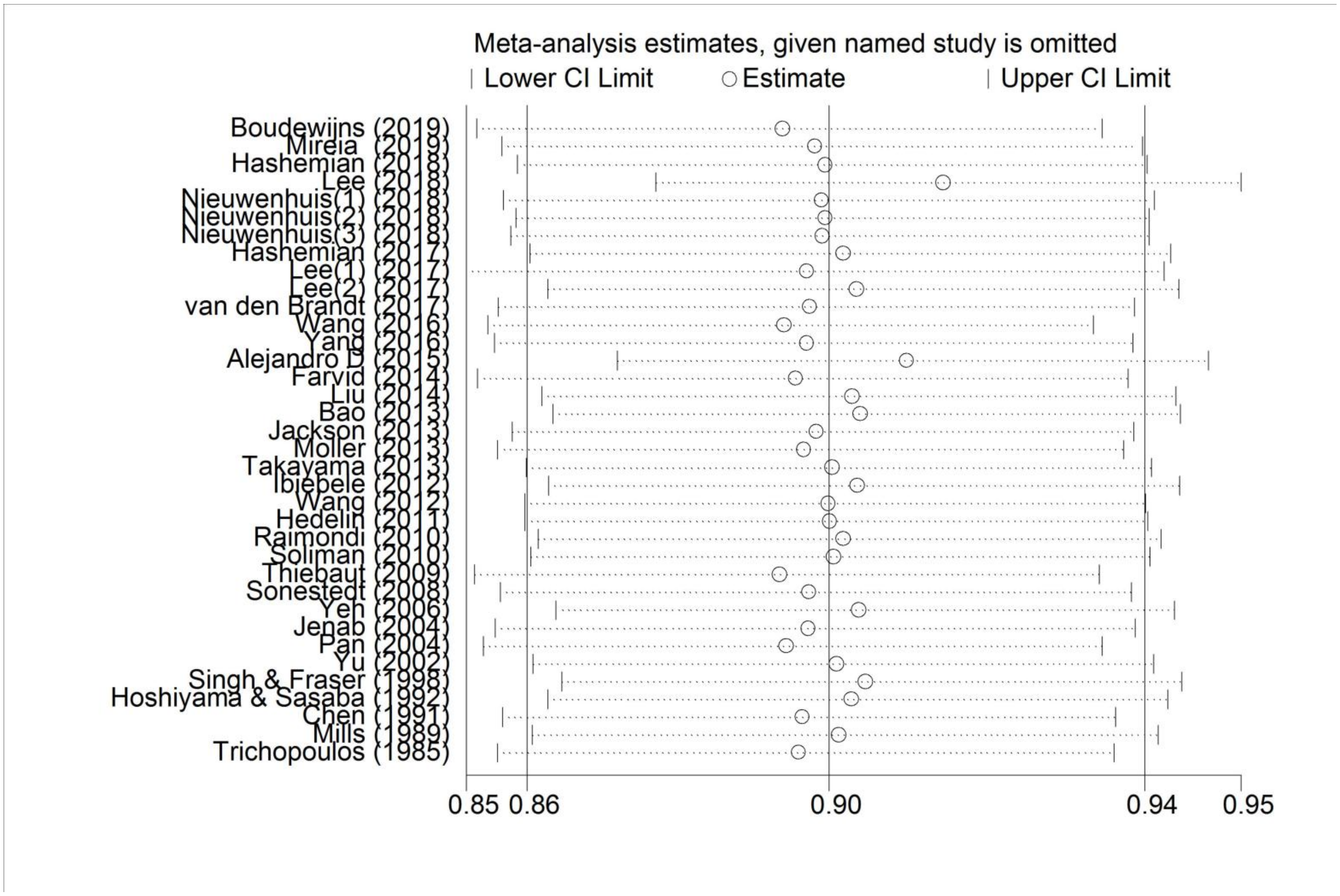

Supplementary Figure 1. The sensitivity analysis of the included studies.

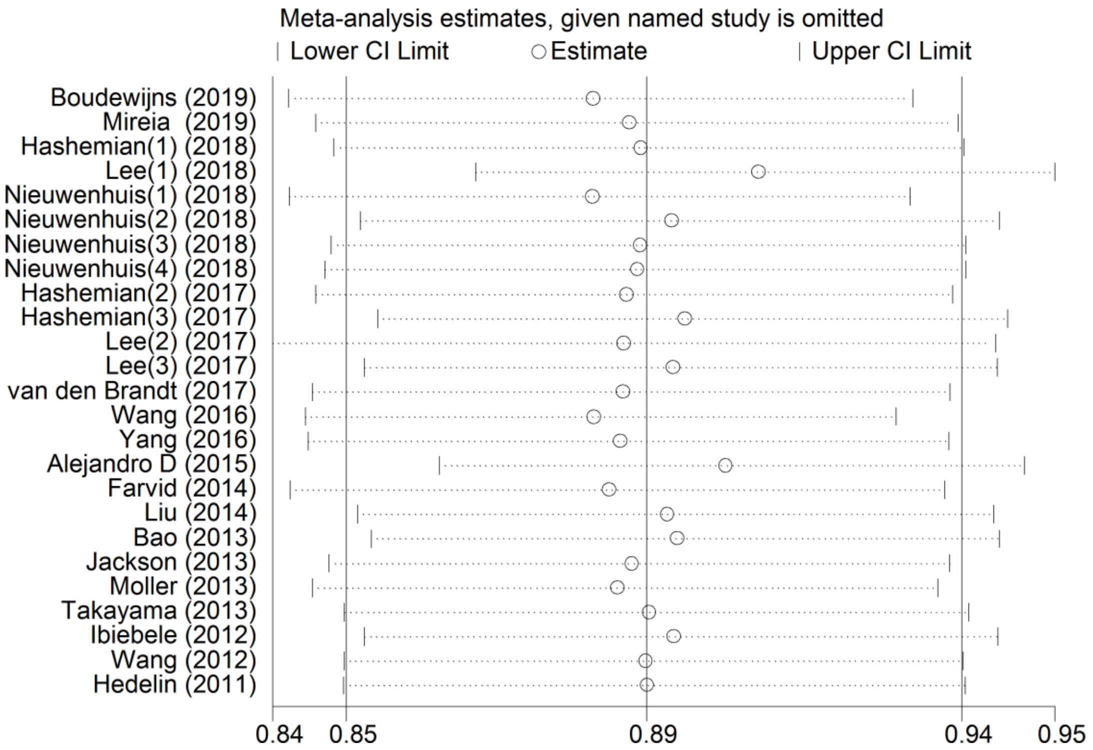

Supplementary Figure 2. The sensitivity analysis of the included studies published after 2010.

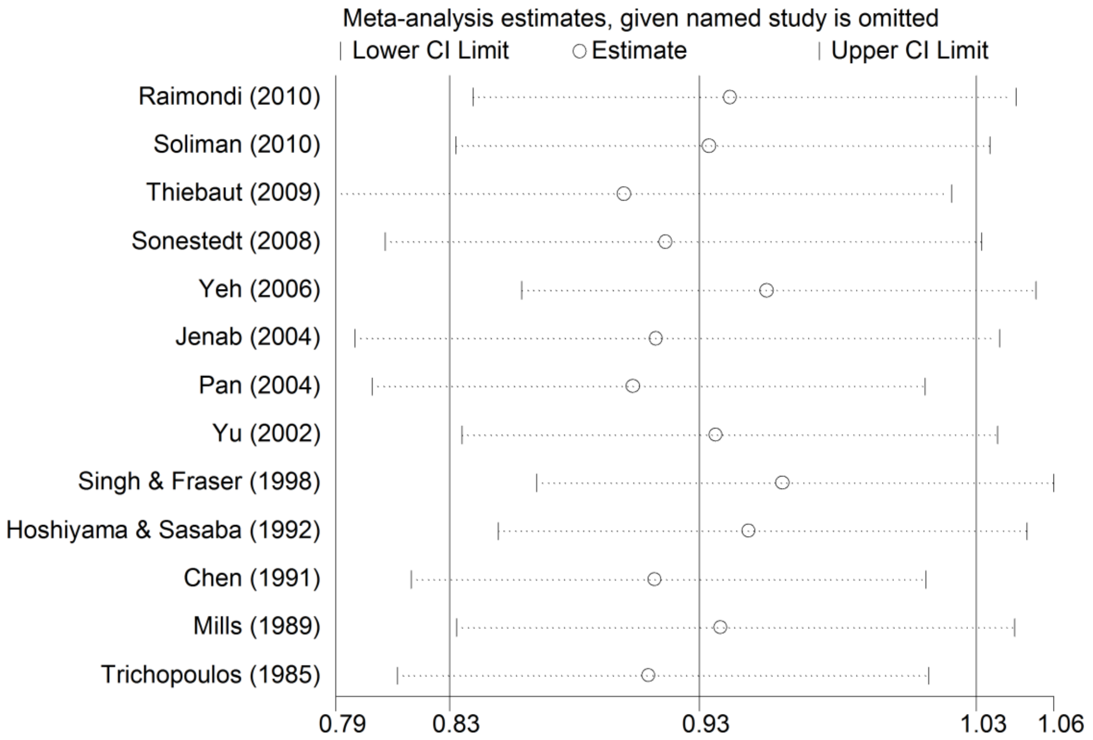

Supplementary Figure 3. The sensitivity analysis of the included studies published in/before 2010.

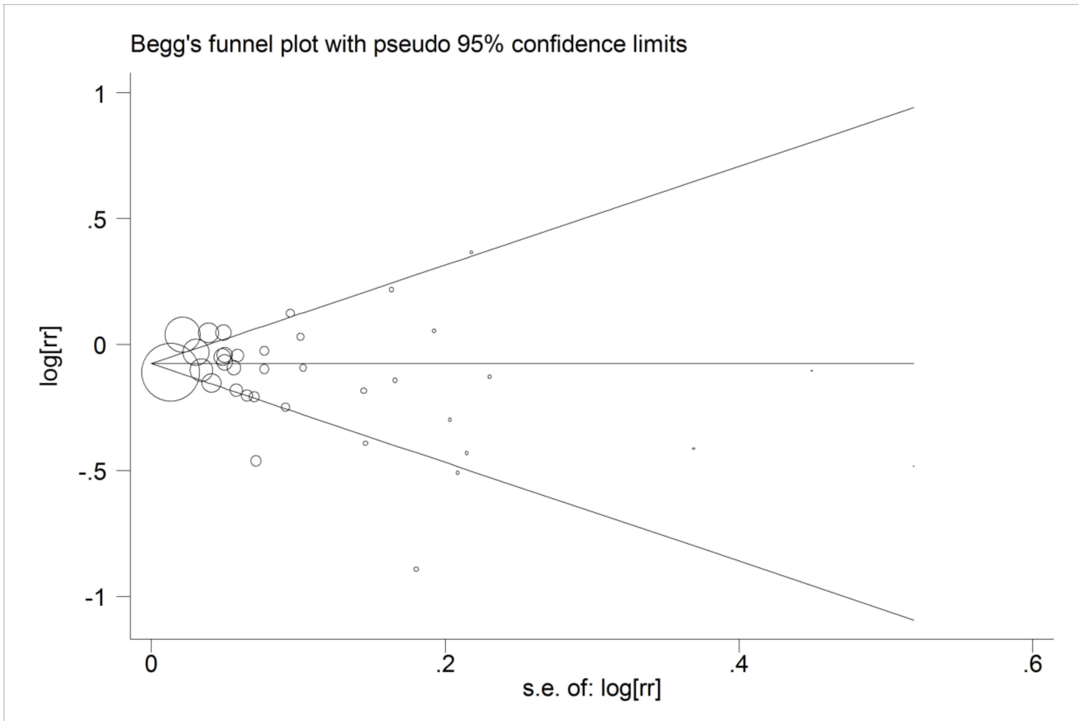

Supplementary Figure 4. Funnel plots with 95% confidence interval of the total studies on cancer risk.

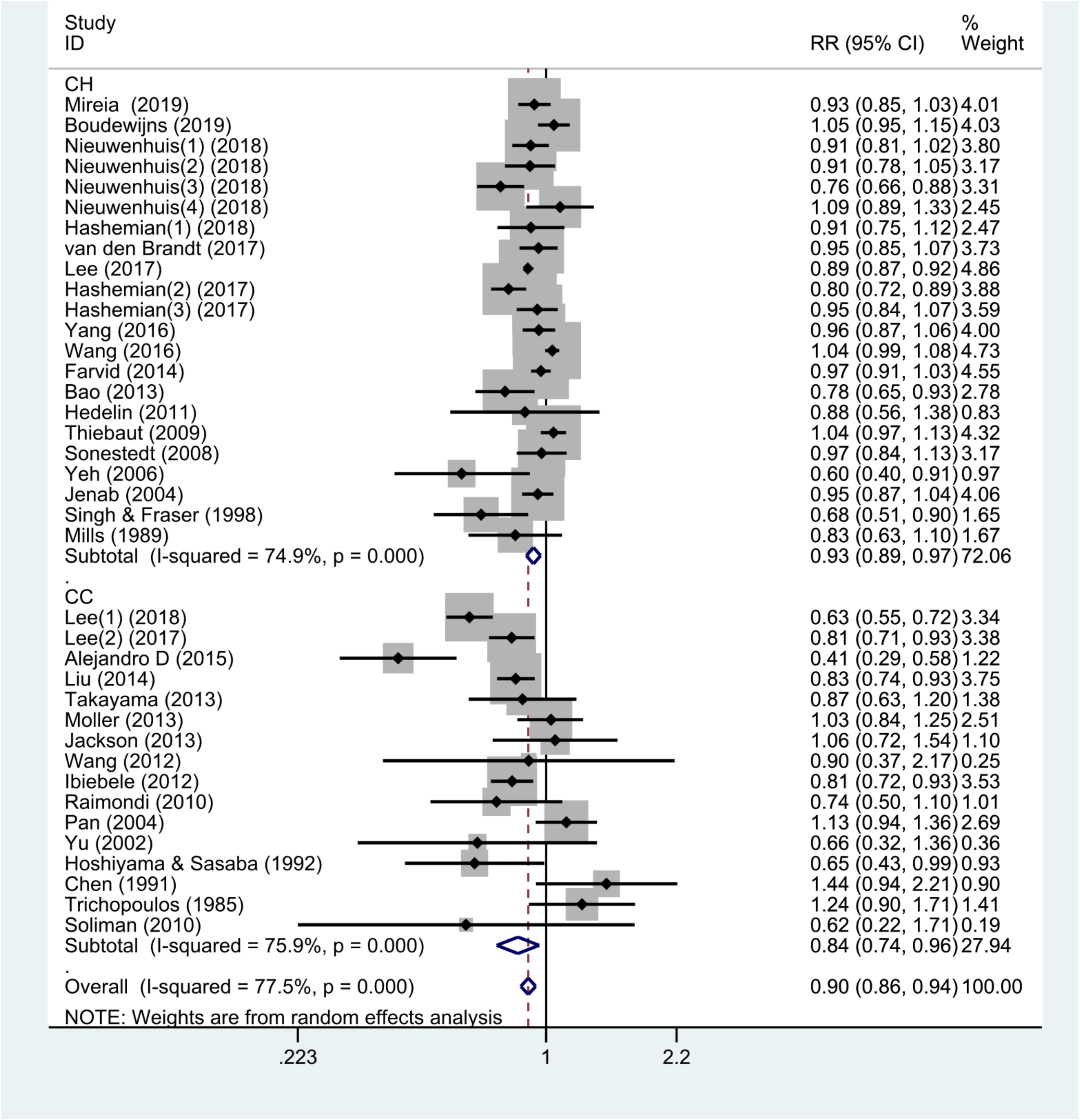

Supplementary Figure 5. Subgroup analyses of the associations between nut intake and cancer risk stratified by study types. Abbreviations: RR, relative risk; CI, confidence interval, CH, cohort study; CC, case-control study.

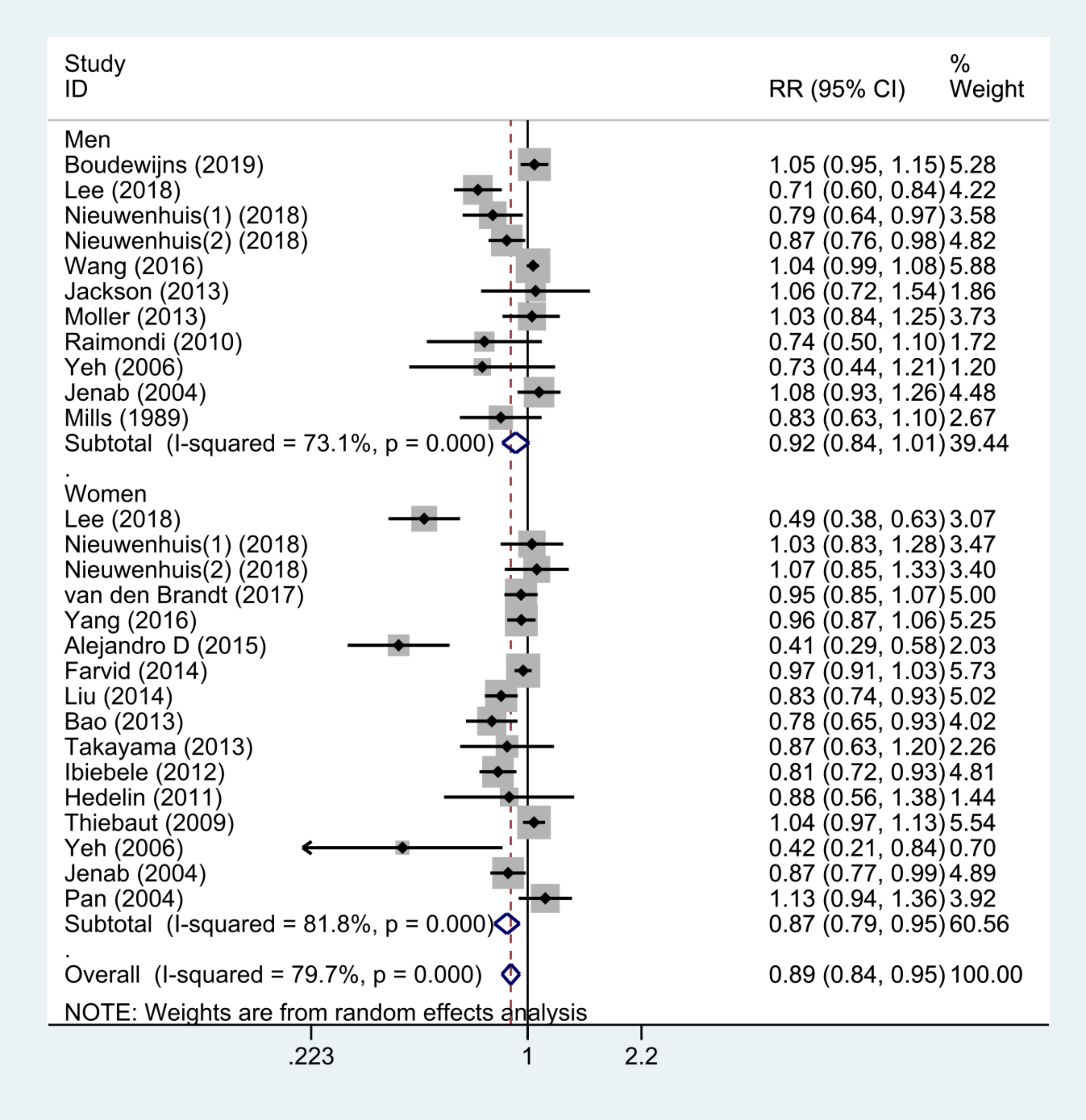

Supplementary Figure 6. Subgroup analyses of the associations between nut intake and cancer risk stratified by gender. Abbreviations: RR, relative risk; CI, confidence interval.

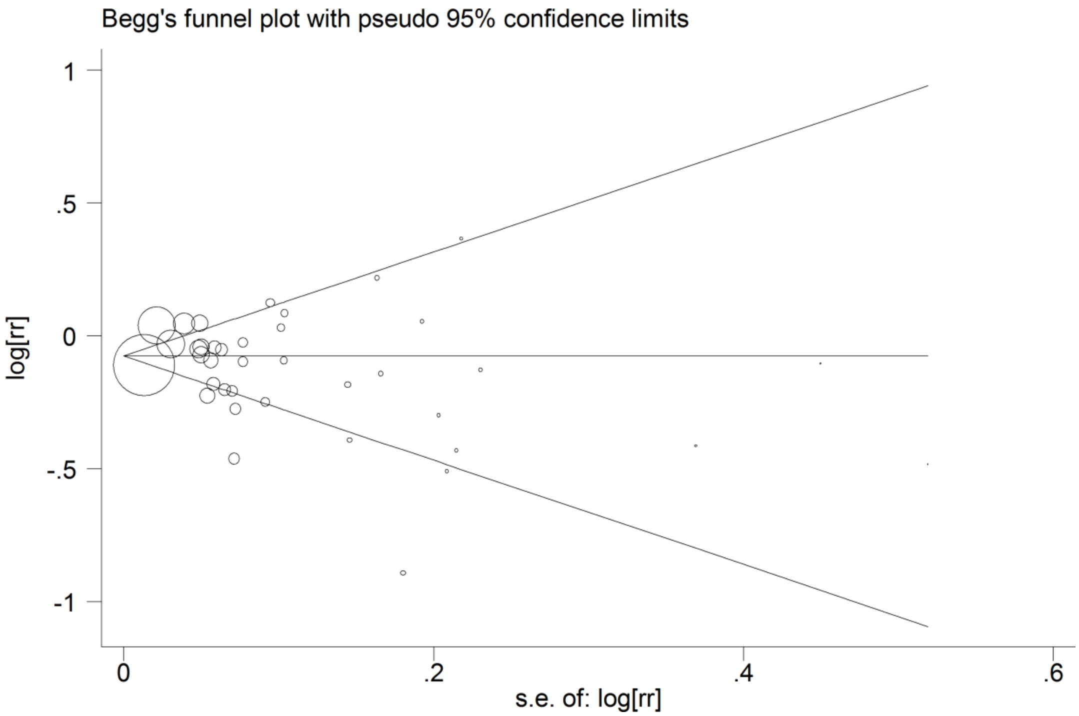

Supplementary Figure 7. Funnel plots with 95% confidence interval of the cancer type subgroup analysis.

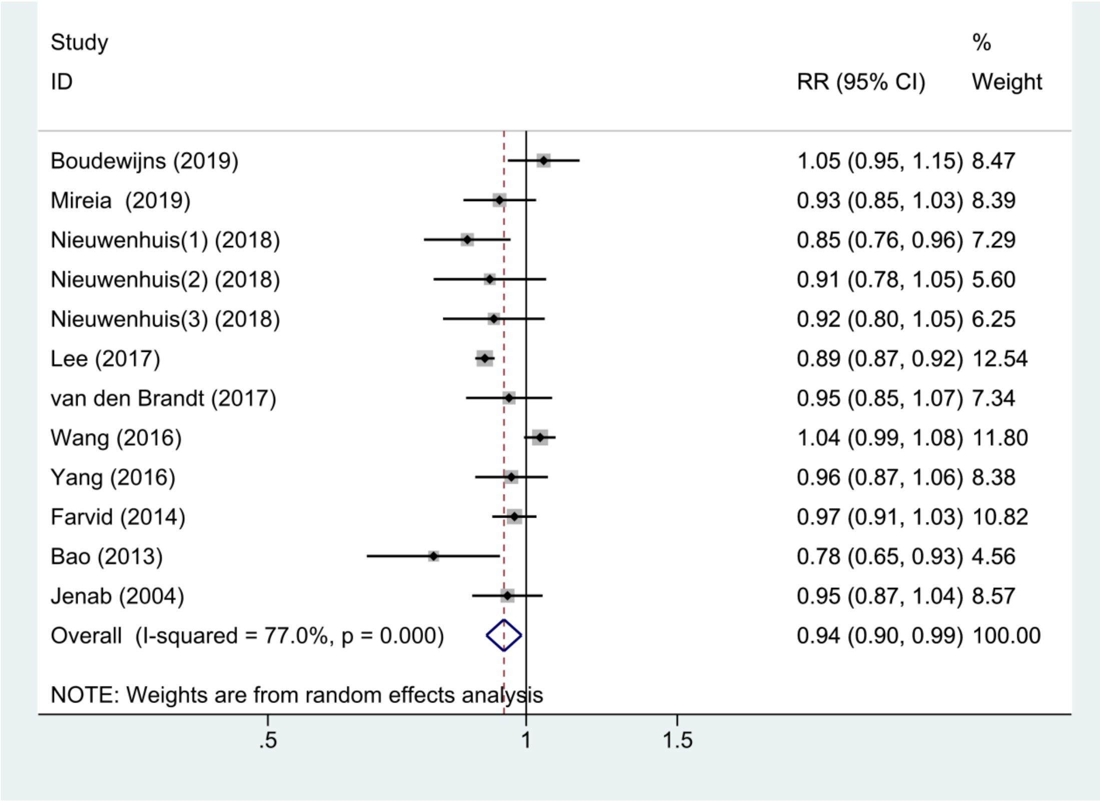

Supplementary Figure 8. Meta-analysis of the studies conducted to assess the dose-response relationship between nut intake and cancer risk. Abbreviations: RR, relative risk; CI, confidence interval.

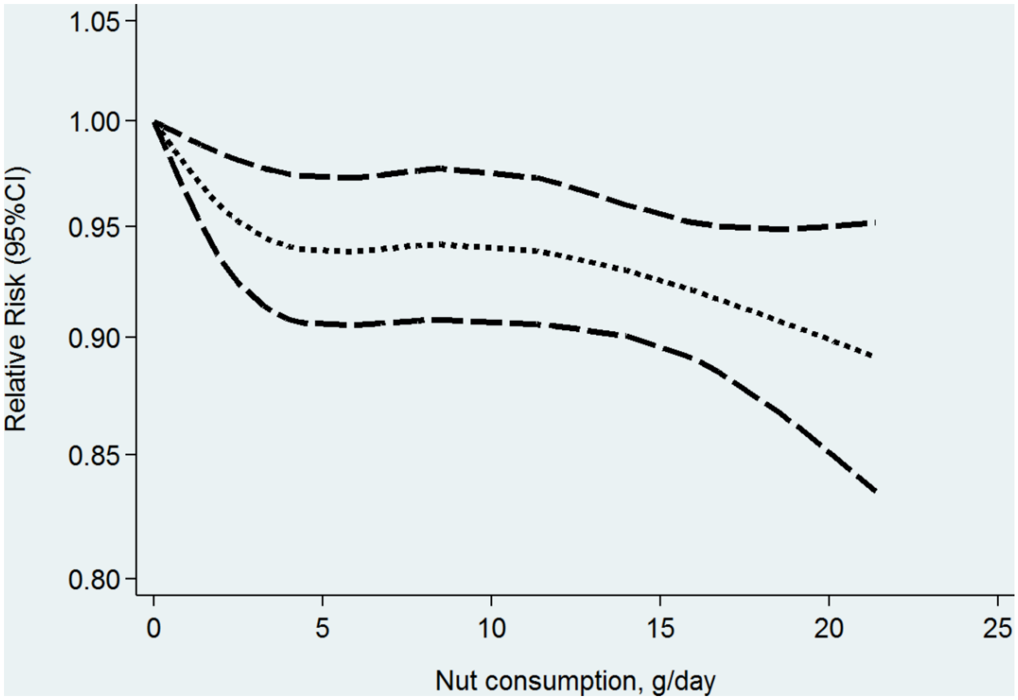

Supplementary Figure 9. Dose–response meta-analysis of nut intake and cancer risk (nonlinear models). Abbreviations: RR, relative risk; CI, confidence interval.

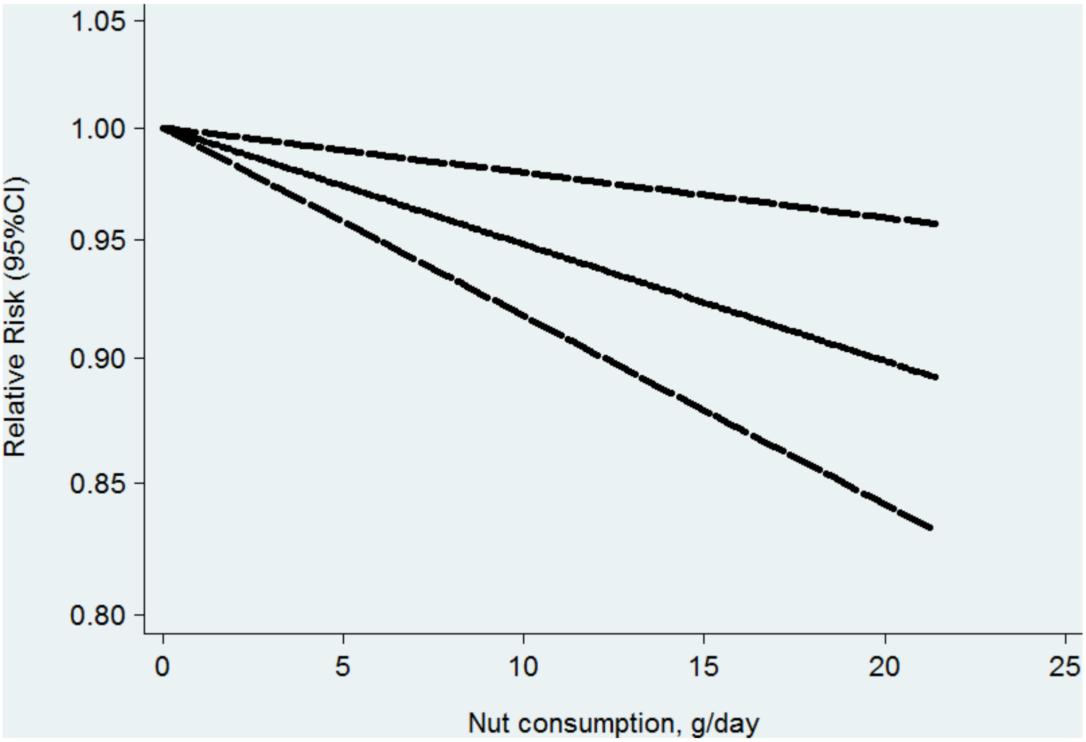

Supplementary Figure 10. Dose–response meta-analysis of nut intake and cancer risk (linear models). Abbreviations: RR, relative risk; CI, confidence interval.
