# Supplementary material for: Meta-analysis of the association between nut consumption and the risks of cancer incidence and cancer-specific mortality
Source: Aging (Albany NY). 2020 Jun 2;12(11):10772–94. doi: 10.18632/aging.103292 (PMC7346045; doi:10.18632/aging.103292)
Supplement: Supplementary Table 1 [file aging-12-103292-s002..doc]

| **Supplementary Table 1. Characteristics of included studies on nut intake and cancer risk in this meta-analysis.** | | | | | | | | | | | |
| --- | --- | --- | --- | --- | --- | --- | --- | --- | --- | --- | --- |
| References | Study type | Follow-up (year) | Country | Age(year) | No .of cases/ no. of overall subjects in CH or no. of controls in CC | Exposure categories (exposure/case assessment) | RR (95%CI) | Monotherapy or Adjusted and Matched/adjusted factors | Outcome investigated | NOS |  |
| Boudewijns | Cohort study | 20.3 | Netherlands | M.55-69 | 3868/58279 | Total nuts(g/d): 0 (Ref) |  | Adjusted：age, family history of prostate cancer, alcohol consumption, level of education (primary school or lower vocational education (low)/secondary school or medium vocational education (medium)/university or higher vocational education (high)), BMI, and total energy intake. | prostate cancer | 9 |  |
| 2019 |  |  |  |  |  | 0.1 to < 5 | 1.05(0.91-1.22) |  |  |  |  |
|  |  |  |  |  |  | 5 to <10 | 0.99(0.81-1.2) |  |  |  |  |
|  |  |  |  |  |  | 10+ | 1.09(0.92-1.29) |  |  |  |  |
|  |  |  |  |  |  | Tree nuts(g/d): 0 (Ref) |  |  |  |  |  |
|  |  |  |  |  |  | 0.1 to < 5 | 1.00(0.87-1.16) |  |  |  |  |
|  |  |  |  |  |  | 5 + | 1.10(0.84-1.44) |  |  |  |  |
|  |  |  |  |  |  | peanuts(g/d): 0 (Ref) |  |  |  |  |  |
|  |  |  |  |  |  | 0.1 to < 5 | 1.07(0.93-1.23) |  |  |  |  |
|  |  |  |  |  |  | 5 to <10 | 1.06(0.86-1.31) |  |  |  |  |
|  |  |  |  |  |  | 10+ | 1.11(0.93-1.33) |  |  |  |  |
|  |  |  |  |  |  | Peanut butter(g/d): 0 (Ref) |  |  |  |  |  |
|  |  |  |  |  |  | 0.1 to < 5 | 1.14(0.97-1.33) |  |  |  |  |
|  |  |  |  |  |  | 5 + | 1.19(0.99-1.44) |  |  |  |  |
| Mireia  2019 | Cohort study | 14 | European | M&F.47-55 | 1283/476160 | Total nuts(g/d): 0 (Ref) |  | Adjusted: age, smoking (never, ever), diabetes (yes, no) and BMI (<25, ≥25 kg/m2). Heavy alcohol consumption (>60, 0.1–4.9 g/day) | Pancreatic Cancer | 9 |  |
|  |  |  |  |  |  | Q1 Median: 0.3 | 0.92(0.74–1.14) |  |  |  |  |
|  |  |  |  |  |  | Q2 Median: 1.0 | 0.92 (0.77–1.09) |  |  |  |  |
|  |  |  |  |  |  | Q3 Median: 3.5 | 0.99 (0.82–1.20) |  |  |  |  |
|  |  |  |  |  |  | Q4 Median: 11.8 | 0.89 (0.72–1.10) |  |  |  |  |
| Hashemian | Cohort study | 9 | Iran | M&F.≥40 | 280/50045 | Total nuts: 0 (Ref) |  | Adjusted: age, sex, BMI, ethnicity, place of residence, smoking status (pack-year), opium use, alcohol drinking (never, ever), physical activity at work, 19 wealth score, intake of fruits and vegetables and total energy intake | Esophageal squamous cell carcinoma | 8 |  |
| 2018 |  |  |  |  |  | Tertile 1 | 1.02(0.75-1.39) |  |  |  |  |
|  |  |  |  |  |  | Tertile 2 | 1.03(0.73-1.44) |  |  |  |  |
|  |  |  |  |  |  | Tertile 3 | 0.60(0.39-0.93) |  |  |  |  |
| Nieuwenhuis  2018 | Cohort study | 20.3 | Netherlands | M&F.55-69 | 1110/120852 | Total nut intake(g/d): 0 (Ref) |  | Adjusted：age at baseline, sex, cigarette smoking ,frequency and duration, BMI, non-occupational physical activity, highest level of education, total energy intake, alcohol consumption and family history of esophageal or gastric cancer | Esophageal squamous cell carcinoma | 8 |  |
|  |  |  |  |  |  | 0.1–< 5 | 1.05(0.66-1.66) |  |  |  |  |
|  |  |  |  |  |  | 5–< 10 | 0.95(0.49-1.84) |  |  |  |  |
| References | Study type | Follow-up (year) | Country | Age(year) | No .of cases/ no. of overall subjects in CH or no. of controls in CC | Exposure categories (exposure/case assessment) | RR (95%CI) | Monotherapy or Adjusted and Matched/adjusted factors | Outcome investigated | NOS |  |
|  |  |  |  |  |  | 10 + | 0.46(0.23-0.94） |  |  |  |  |
|  |  |  |  |  |  | Tree nuts(g/d): 0 (Ref) |  |  |  |  |  |
|  |  |  |  |  |  | 0.1+ | 0.67(0.41-1.09) |  |  |  |  |
|  |  |  |  |  |  | peanuts (g/d): 0 (Ref) |  |  |  |  |  |
|  |  |  |  |  |  | 0.1–< 5 | 1.04(0.67-1.63) |  |  |  |  |
|  |  |  |  |  |  | 5–< 10 | 1.06(0.53-2.13) |  |  |  |  |
|  |  |  |  |  |  | 10 + | 0.46(0.21-1.00) |  |  |  |  |
|  |  |  |  |  |  | Peanut butter(g/d): 0 (Ref) |  |  |  |  |  |
|  |  |  |  |  |  | 0.1–< 5 | 0.92(0.53-1.63) |  |  |  |  |
|  |  |  |  |  |  | 5+ | 1.55(0.82-2.93) |  |  |  |  |
|  |  |  |  |  |  | Total nut intake(g/d): 0 (Ref) |  |  | Esophageal adenocarcinoma | 8 |  |
|  |  |  |  |  |  | 0.1–< 5 | 1.23(0.83-1.81) |  |  |  |  |
|  |  |  |  |  |  | 5–< 10 | 1.41(0.85-2.34) |  |  |  |  |
|  |  |  |  |  |  | 10 + | 1.19(0.74-1.91) |  |  |  |  |
|  |  |  |  |  |  | Tree nuts(g/d): 0 (Ref) |  |  |  |  |  |
|  |  |  |  |  |  | 0.1+ | 1.04(0.74-1.48) |  |  |  |  |
|  |  |  |  |  |  | peanuts(g/d): 0 (Ref) |  |  |  |  |  |
|  |  |  |  |  |  | 0.1–< 5 | 1.14(0.78-1.67) |  |  |  |  |
|  |  |  |  |  |  | 5–< 10 | 1.31(0.75-2.29) |  |  |  |  |
|  |  |  |  |  |  | 10 + | 1.19(0.72-1.97) |  |  |  |  |
|  |  |  |  |  |  | Peanut butter(g/d): 0 (Ref) |  |  |  |  |  |
|  |  |  |  |  |  | 0.1–< 5 | 1.29(0.87-1.92) |  |  |  |  |
|  |  |  |  |  |  | 5+ | 1.10(0.65-1.87) |  |  |  |  |
|  |  |  |  |  |  | Total nut intake(g/d): 0 (Ref) |  |  | Gastric cardia adenocarcinoma | 8 |  |
|  |  |  |  |  |  | 0.1–< 5 | 1.05(0.7-1.58) |  |  |  |  |
|  |  |  |  |  |  | 5–< 10 | 0.99(0.56-1.76) |  |  |  |  |
|  |  |  |  |  |  | 10 + | 0.91(0.56-1.49) |  |  |  |  |
|  |  |  |  |  |  | Tree nuts(g/d): 0 (Ref) |  |  |  |  |  |
| References | Study type | Follow-up (year) | Country | Age(year) | No .of cases/ no. of overall subjects in CH or no. of controls in CC | Exposure categories (exposure/case assessment) | RR (95%CI) | Monotherapy or Adjusted and Matched/adjusted factors | Outcome investigated | NOS |  |
|  |  |  |  |  |  | 0.1+ | 0.95(0.64-1.4) |  |  |  |  |
|  |  |  |  |  |  | peanuts(g/d): 0 (Ref) |  |  |  |  |  |
|  |  |  |  |  |  | 0.1–< 5 | 0.95(0.64-1.4) |  |  |  |  |
|  |  |  |  |  |  | 5–< 10 | 0.90(0.49-1.64) |  |  |  |  |
|  |  |  |  |  |  | 10 + | 1.01(0.61-1.67) |  |  |  |  |
|  |  |  |  |  |  | Peanut butter(g/d): 0 (Ref) |  |  |  |  |  |
|  |  |  |  |  |  | 0.1–< 5 | 1.15(0.76-1.75) |  |  |  |  |
|  |  |  |  |  |  | 5+ | 0.65(0.34-1.25) |  |  |  |  |
|  |  |  |  |  |  | Total nut intake(g/d): 0 (Ref) |  |  | Gastric non-cardia adenocarcinoma | 8 |  |
|  |  |  |  |  |  | 0.1–< 5 | 0.75(0.59-0.95) |  |  |  |  |
|  |  |  |  |  |  | 5–< 10 | 0.63(0.44-0.9) |  |  |  |  |
|  |  |  |  |  |  | 10 + | 0.64(0.47-0.87) |  |  |  |  |
|  |  |  |  |  |  | Tree nuts(g/d): 0 (Ref) |  |  |  |  |  |
|  |  |  |  |  |  | 0.1+ | 0.84(0.66-1.06) |  |  |  |  |
|  |  |  |  |  |  | peanuts(g/d): 0 (Ref) |  |  |  |  |  |
|  |  |  |  |  |  | 0.1–< 5 | 0.79(0.62-0.99) |  |  |  |  |
|  |  |  |  |  |  | 5–< 10 | 0.63(0.45-0.99) |  |  |  |  |
|  |  |  |  |  |  | 10 + | 0.73(0.52-1.01) |  |  |  |  |
|  |  |  |  |  |  | Peanut butter(g/d): 0 (Ref) |  |  |  |  |  |
|  |  |  |  |  |  | 0.1–< 5 | 1.09(0.84-1.42) |  |  |  |  |
|  |  |  |  |  |  | 5+ | 0.83(0.58-1.18) |  |  |  |  |
| Nieuwenhuis | Cohort study | 20.3 | Netherlands | M&F.55-69 | 583/120852 | Males: |  | Adjusted： age (years; continuous); sex (men/women; in the analyses for the total population); cigarette smoking [status (never/former/current), frequency (n/d; continuous, centered), and duration, body mass index (BMI; kg/m2; continuous); family history of pancreatic cancer (no/yes); history of diabetes, educational level, total energy intake (kcal/d; continuous) and alcohol consumption (g/d; continuous). | Pancreatic Cancer | 8 |  |
| 2018 |  |  |  |  |  | Total nuts(g/d): 0 (Ref) |  |  |  |  |  |
|  |  |  |  |  |  | 0.1 to < 5 | 0.85(0.62-1.17) |  |  |  |  |
|  |  |  |  |  |  | 5 to <10 | 0.78(0.5-1.19) |  |  |  |  |
|  |  |  |  |  |  | 10+ | 0.71(0.5-1.03) |  |  |  |  |
|  |  |  |  |  |  | Tree nuts(g/d): 0 (Ref) |  |  |  |  |  |
|  |  |  |  |  |  | 0.1 to < 5 | 0.68(0.48-0.95) |  |  |  |  |
|  |  |  |  |  |  | 5 + | 0.66(0.34-1.26) |  |  |  |  |
| References | Study type | Follow-up (year) | Country | Age(year) | No .of cases/ no. of overall subjects in CH or no. of controls in CC | Exposure categories (exposure/case assessment) | RR (95%CI) | Monotherapy or Adjusted and Matched/adjusted factors | Outcome investigated | NOS |  |
|  |  |  |  |  |  | peanuts(g/d): 0 (Ref) |  |  |  |  |  |
|  |  |  |  |  |  | 0.1 to < 5 | 0.92(0.68-1.24) |  |  |  |  |
|  |  |  |  |  |  | 5 to <10 | 0.77(0.49-1.21) |  |  |  |  |
|  |  |  |  |  |  | 10+ | 0.82(0.56-1.19) |  |  |  |  |
|  |  |  |  |  |  | Peanut butter(g/d): 0 (Ref) |  |  |  |  |  |
|  |  |  |  |  |  | 0.1 to < 5 | 1.24(0.89-1.72) |  |  |  |  |
|  |  |  |  |  |  | 5 + | 0.66(0.41-1.08) |  |  |  |  |
|  |  |  |  |  |  | Females: |  |  |  |  |  |
|  |  |  |  |  |  | Total nuts(g/d): 0 (Ref) |  |  |  |  |  |
|  |  |  |  |  |  | 0.1 to < 5 | 1.11(0.83-1.49) |  |  |  |  |
|  |  |  |  |  |  | 5 to <10 | 0.89(0.54-1.44) |  |  |  |  |
|  |  |  |  |  |  | 10+ | 0.98(0.63-1.5) |  |  |  |  |
|  |  |  |  |  |  | Tree nuts(g/d): 0 (Ref) |  |  |  |  |  |
|  |  |  |  |  |  | 0.1 to < 5 | 1.02(0.74-1.39) |  |  |  |  |
|  |  |  |  |  |  | 5 + | 0.63(0.32-1.24) |  |  |  |  |
|  |  |  |  |  |  | peanuts(g/d): 0 (Ref) |  |  |  |  |  |
|  |  |  |  |  |  | 0.1 to < 5 | 1.05(0.79-1.4) |  |  |  |  |
|  |  |  |  |  |  | 5 to <10 | 1.11(0.67-1.84) |  |  |  |  |
|  |  |  |  |  |  | 10+ | 0.94(0.57-1.55) |  |  |  |  |
|  |  |  |  |  |  | Peanut butter(g/d): 0 (Ref) |  |  |  |  |  |
|  |  |  |  |  |  | 0.1 to < 5 | 1.11(0.79-1.55) |  |  |  |  |
|  |  |  |  |  |  | 5 + | 0.86(0.53-1.39) |  |  |  |  |
| Nieuwenhuis | Cohort study | 20.3 | Netherlands | M&F.55-69 | 2861/120852 | Males: |  | Adjusted： age, smoking, body mass index Non-occupational physical activity, educational level, family history of lung cancer (yes, no), history of chronic bronchitis (yes, no), daily energy intake (kcal/day; continuous), alcohol consumption, and alternate Mediterranean (aMED) diet score excluding alcohol and nuts (0–2, 3–4, 5–7 points). | Lung cancer | 9 |  |
| 2018 |  |  |  |  |  | Total nuts(g/d): 0 (Ref) |  |  |  |  |  |
|  |  |  |  |  |  | 0.1 to < 5 | 0.87(0.72-1.06) |  |  |  |  |
|  |  |  |  |  |  | 5 to <10 | 0.91(0.7-1.19) |  |  |  |  |
|  |  |  |  |  |  | 10+ | 0.83(0.67-1.04) |  |  |  |  |
|  |  |  |  |  |  | Tree nuts(g/d): 0 (Ref) |  |  |  |  |  |
|  |  |  |  |  |  | 0.1+ | 0.87(0.72-1.04) |  |  |  |  |
| References | Study type | Follow-up (year) | Country | Age(year) | No .of cases/ no. of overall subjects in CH or no. of controls in CC | Exposure categories (exposure/case assessment) | RR (95%CI) | Monotherapy or Adjusted and Matched/adjusted factors | Outcome investigated | NOS |  |
|  |  |  |  |  |  | peanuts(g/d): 0 (Ref) |  |  |  |  |  |
|  |  |  |  |  |  | 0.1 to < 5 | 0.88(0.73-1.06) |  |  |  |  |
|  |  |  |  |  |  | 5 + | 0.89(0.72-1.08) |  |  |  |  |
|  |  |  |  |  |  | Peanut butter(g/d): 0 (Ref) |  |  |  |  |  |
|  |  |  |  |  |  | 0.1 to < 5 | 0.98(0.79-1.2) |  |  |  |  |
|  |  |  |  |  |  | 5 + | 0.92(0.7-1.19) |  |  |  |  |
|  |  |  |  |  |  | Females: |  |  |  |  |  |
|  |  |  |  |  |  | Total nuts(g/d): 0 (Ref) |  |  |  |  |  |
|  |  |  |  |  |  | 0.1 to < 5 | 1.10(0.81-1.49) |  |  |  |  |
|  |  |  |  |  |  | 5 to <10 | 1.17(0.73-1.87） |  |  |  |  |
|  |  |  |  |  |  | 10+ | 0.91(0.58-1.43) |  |  |  |  |
|  |  |  |  |  |  | Tree nuts(g/d): 0 (Ref) |  |  |  |  |  |
|  |  |  |  |  |  | 0.1+ | 1.01(0.74-1.37) |  |  |  |  |
|  |  |  |  |  |  | peanuts(g/d): 0 (Ref) |  |  |  |  |  |
|  |  |  |  |  |  | 0.1 to < 5 | 1.15(0.86-1.55) |  |  |  |  |
|  |  |  |  |  |  | 5 + | 1.05(0.7-1.56) |  |  |  |  |
|  |  |  |  |  |  | Peanut butter(g/d): 0 (Ref) |  |  |  |  |  |
|  |  |  |  |  |  | 0.1 to < 5 | 1.03(0.72-1.45) |  |  |  |  |
|  |  |  |  |  |  | 5 + | 1.03(0.65-1.62) |  |  |  |  |
| Hashemian | Cohort study | 15.5 | American | M&F.50-71 | 2671/566407 | Categories of nut intake:0 (Ref) |  | Adjusted： age, sex, smoking status and dose, alcohol drinking (grams per day), BMI (kg/m2), education, leisure physical activity, fruit and vegetable intake (servings per day), and calorie intake (kilocalories per day). energy intake, Ethnicity |  |  |  |
| 2017 |  |  |  |  |  | C1 | 0.94(0.73-1.21) |  | Esophageal adenocarcinoma | 8 |  |
|  |  |  |  |  |  | C2 | 0.97(0.76-1.24) |  |  |  |  |
|  |  |  |  |  |  | C3 | 0.90(0.7-1.16) |  |  |  |  |
|  |  |  |  |  |  | C1 | 1.00(0.66-1.53) |  | Esophageal squamous cell carcinoma | 8 |  |
|  |  |  |  |  |  | C2 | 0.93(0.61-1.44) |  |  |  |  |
|  |  |  |  |  |  | C3 | 1.01(0.66-1.55) |  |  |  |  |
|  |  |  |  |  |  | C1 | 0.71(0.54-0.94) |  | Gastric cardia adenocarcinoma | 8 |  |
|  |  |  |  |  |  | C2 | 0.76(0.58-1.00) |  |  |  |  |
|  |  |  |  |  |  | C3 | 0.88(0.67-1.150 |  |  |  |  |
| References | Study type | Follow-up (year) | Country | Age(year) | No .of cases/ no. of overall subjects in CH or no. of controls in CC | Exposure categories (exposure/case assessment) | RR (95%CI) | Monotherapy or Adjusted and Matched/adjusted factors | Outcome investigated | NOS |  |
|  |  |  |  |  |  | C1 | 0.93(0.73-1.18) |  | Gastric non-cardia adenocarcinoma | 8 |  |
|  |  |  |  |  |  | C2 | 0.77(0.6-0.99) |  |  |  |  |
|  |  |  |  |  |  | C3 | 0.73(0.57-0.94) |  |  |  |  |
| Lee | Cohort study | 16 | American | M&F.35-79 | 18533/495785 | Quintile of total nuts:1: 0 (Ref) |  | Adjusted： age, sex, and cigarette smoking dose, body mass index (BMI), education, cigarette smoking status, and years since last cigarette smoked for former-smokers energy intake. fruits, vegetables, red and processed meat, and alcohol. | Lung cancer | 8 |  |
| 2017 |  |  |  |  |  | 2(1–6 times a year) | 0.93(0.89-0.98) |  |  |  |  |
|  |  |  |  |  |  | 3(7–11 times a year) | 0.89(0.85-0.94) |  |  |  |  |
|  |  |  |  |  |  | 4(1-3times/month | 0.89(0.84-0.94) |  |  |  |  |
|  |  |  |  |  |  | 5 1-6 Times/week | 0.86(0.81-0.91) |  |  |  |  |
| Brandt | Cohort study | 20.3 | Netherlands | F.55-69 | 2321/62573 | Total nut intake(g/d): 0 (Ref) |  | Adjusted： age at baseline， cigarette smoking status, frequency, duration (number of years; continuous, centered), body height, BMI non-occupational physical activity, highest level of education, family history of breast cancer in mother or sisters (no, yes), history of benign breast disease (no, yes), age at menarche, parity, age at first birth age at menopause, oral contraceptive use, postmenopausal hormone replacement therapy, energy intake (continuous, kcal/day), alcohol intake. | Breast cancer | 8 |  |
| 2017 |  |  |  |  |  | 0.1–< 5 | 0.94(0.8-1.1) |  |  |  |  |
|  |  |  |  |  |  | 5–< 10 | 1.05(0.82-1.34) |  |  |  |  |
|  |  |  |  |  |  | 10 + | 0.91(0.72-1.14) |  |  |  |  |
|  |  |  |  |  |  | peanuts(g/d): 0 (Ref) |  |  |  |  |  |
|  |  |  |  |  |  | 0.1–< 5 | 1.00(0.86–1.17) |  |  |  |  |
|  |  |  |  |  |  | 5–< 10 | 0.98(0.79–1.20) |  |  |  |  |
|  |  |  |  |  |  | Tree nuts(g/d): 0 (Ref) |  |  |  |  |  |
|  |  |  |  |  |  | 0.1–< 5 | 1.00(0.84–1.18) |  |  |  |  |
|  |  |  |  |  |  | 5–< 10 | 0.80(0.58–1.10) |  |  |  |  |
|  |  |  |  |  |  | Peanut butter(g/d): 0 (Ref) |  |  |  |  |  |
|  |  |  |  |  |  | 0.1–< 5 | 1.05(0.88–1.26) |  |  |  |  |
|  |  |  |  |  |  | 5–< 10 | 1.01(0.79–1.29) |  |  |  |  |
| Wang | Cohort study | 26 | American | M.NA | 6810/47299 | Total nut intake:<once/month(Ref) |  | Adjusted：age in months, time period (2-year intervals), energy body mass index, vigorous physical activity, smoking status, and PSA screening history. family history of PCa (yes, no), ethnicity height, history of diabetes (yes, no), current multivitamin use (yes, no), current supplement use (yes, no), tomato sauce, coffee intake, and Mediterranean diet. | Prostate cancer | 7 |  |
| 2016 |  |  |  |  |  | <Once/week | 1.04(0.96-1.13) |  |  |  |  |
|  |  |  |  |  |  | Once per week | 1.06(0.98-1.15) |  |  |  |  |
|  |  |  |  |  |  | 2–4 Times/week | 1.05(0.97-1.14) |  |  |  |  |
|  |  |  |  |  |  | ≥5 Times/week | 0.98(0.89-1.09) |  |  |  |  |
| Yang | Cohort study | 30 | American | F.30-55 | 1503/75680 | Total nut intake: never (Ref) |  | Adjusted: age (month), physical activity family history of colorectal cancer (yes/no), history of previous lower endoscopy | Colorectal cancer | 7 |  |
| 2016 |  |  |  |  |  | 1–3 times/month | 1.00(0.86-1.16) |  |  |  |  |
| References | Study type | Follow-up (year) | Country | Age(year) | No .of cases/ no. of overall subjects in CH or no. of controls in CC | Exposure categories (exposure/case assessment) | RR (95%CI) | Monotherapy or Adjusted and Matched/adjusted factors | Outcome investigated | NOS |  |
|  |  |  |  |  |  | Once/week | 0.98(0.82-1.17) | (yes/no), history of ulcerative colitis, history of polyps (yes/no), aspirin use, multivitamin use (yes/no), pack-years of smoking, alcohol intake and total energy intake, body-mass index and history of diabetes mellitus (yes/no). |  |  |  |
|  |  |  |  |  |  | ≥ 2 times/week | 0.87(0.72-1.05) |  |  |  |  |
| Bao | Cohort study | 30 | American | F.30-55 | 466/75 680 | Nut consumption: never (Ref) |  | Adjusted： age, height, physical activity, smoking, total energy intake, BMI, history of diabetes mellitus, alcohol consumption, multivitamin use, and intakes of red meat, fruits and vegetables, and vitamin D | Pancreatic cancer | 7 |  |
| 2013 |  |  |  |  |  | 1–3 times/month | 0.90(0.69-1.18) |  |  |  |  |
|  |  |  |  |  |  | Once/week | 0.71(0.51-0.99) |  |  |  |  |
|  |  |  |  |  |  | ≥ 2 times/week | 0.68(0.48-0.96) |  |  |  |  |
| Hedelin | Cohort study | median 16 | Sweden | F.30-49 | 163/47 140 | Nut intake: lowest category (Ref) |  | Adjusted： age, use of oral contraceptives, age at menarche, parity, hormone replacement therapy, and intakes of total energy, alcohol, saturated fat, meat, and fish | Ovarian cancer | 7 |  |
| 2011 |  |  |  |  |  | Highest category | 0.88(0.56–1.38 |  |  |  |  |
| Thiebaut | Cohort study | mean 8 | France | F.40-65 | 1650/56 007 | Quintile of 7.2% linoleic acid (from nuts) |  | Adjusted：age, non-alcohol energy and ethanol intakes, smoking history, history of benign breast disease, history of breast cancer in first degree relatives, age at menarche, parity, BMI, menopausal status, age at menopause, and use of menopausal hormone treatment | Breast cancer | 7 |  |
| 2009 |  |  |  |  |  | Quintile I-0 (Ref) |  |  |  |  |  |
|  |  |  |  |  |  | II | 0.92(0.79–1.08) |  |  |  |  |
|  |  |  |  |  |  | III | 1.01(0.87–1.18) |  |  |  |  |
|  |  |  |  |  |  | IV | 1.09(0.94–1.27) |  |  |  |  |
|  |  |  |  |  |  | V | 1.17(1.01–1.37) |  |  |  |  |
| Sonestedt | Cohort study | mean 10.3 | Sweden | F.46-75 | 544/15 773 | Tertiles of nut intake: no intake (Ref) |  | Adjusted： season of data collection, diet interviewer, method version, age, total energy, weight, height, educational status, smoking habits, leisure-time physical activity, hours of household activities, alcohol consumption, age at menopause, parity, and current use of menopausal hormone therapy | Breast cancer | 9 |  |
| 2008 |  |  |  |  |  | 1 | 0.94(0.72-1.22) |  |  |  |  |
|  |  |  |  |  |  | 2 | 1.00(0.77-1.29) |  |  |  |  |
|  |  |  |  |  |  | 3 | 0.98(0.75-1.27) |  |  |  |  |
| Yeh | Cohort study | 10 | China | M&F.30-65 | 107/23 943 | Peanut intake: 0–1 meal/week (Ref |  | Adjusted： age, area of residence, cigarette smoking, BMI | Colorectal cancer | 8 |  |
| 2006 |  |  |  |  |  | ≥2 meals/week |  |  |  |  |  |
|  |  |  |  |  |  | Men | 0.73(0.44–1.21) |  |  |  |  |
|  |  |  |  |  |  | Women | 0.42(0.21–0.84 |  |  |  |  |
| Farvid | Cohort study | 20 | American | F.24-43 | 2830/88 803 | Nuts intake: Category 1-no intake (Ref) |  | Adjusted： age, race, family history of breast cancer in mother or sisters, history of benign breast disease, smoking, height, BMI, age at menarche, parity and age at first birth, oral | Breast cancer | 7 |  |
| 2014 |  |  |  |  |  | C2(median 0.07 serving/d) | 1.03(0.92–1.15) |  |  |  |  |
|  |  |  |  |  |  | C3(median 0.14 serving/d) | 0.94(0.83–1.06) |  |  |  |  |
| References | Study type | Follow-up (year) | Country | Age(year) | No .of cases/ no. of overall subjects in CH or no. of controls in CC | Exposure categories (exposure/case assessment) | RR (95%CI) | Monotherapy or Adjusted and Matched/adjusted factors | Outcome investigated | NOS |  |
|  |  |  |  |  |  | C4(median 0.21 serving/d) | 0.96(0.85–1.09) | contraceptive use, alcohol intake, and energy intake |  |  |  |
|  |  |  |  |  |  | C5(median 0.57 serving/d) | 0.94(0.83–1.05) |  |  |  |  |
| Jenab  2004 | Cohort study | mean 4.8 | European | M.35–70 | 542/141 988 | Nuts/seeds intake: category 1-never(Ref) |  | Adjusted： Center, age, height, weight, intake of fruits (without nuts and seeds), intake of dietary fiber, physical activity, duration of smoking, gender, energy from alcohol, energy from fat, and energy from carbohydrates and proteins | Colorectal cancer | 8 |  |
|  |  |  |  | F.35–70 | 787/336 052 | Males: |  |  |  |  |  |
|  |  |  |  |  |  | Category 2:<0.8 g/d | 1.10(0.78–1.54) |  |  |  |  |
|  |  |  |  |  |  | Category 3: 0.8–2.3 g/d | 1.03(0.79–1.34) |  |  |  |  |
|  |  |  |  |  |  | Category 4: 2.3–6.2 g/d | 1.13(0.84–1.53) |  |  |  |  |
|  |  |  |  |  |  | Category 5:>6.2 g/d | 1.09(0.81–1.49) |  |  |  |  |
|  |  |  |  |  |  | Females: |  |  |  |  |  |
|  |  |  |  |  |  | Category 2:<0.8 g/d | 0.87(0.64–1.15) |  |  |  |  |
|  |  |  |  |  |  | Category 3: 0.8–2.3 g/d | 0.96(0.77–1.20) |  |  |  |  |
|  |  |  |  |  |  | Category 4: 2.3–6.2 g/d | 0.84(0.67–1.07) |  |  |  |  |
|  |  |  |  |  |  | Category 5:>6.2 g/d | 0.81(0.63–1.04) |  |  |  |  |
|  |  |  |  |  |  | Nuts/seeds intake: category 1-never (Ref) |  | Adjusted： BMI, education, smoking, alcohol intake,  physical activity, and total energy intake  stratified by sex, center, and age at  recruitment | Colon cancer | 8 |  |
|  |  |  |  | M.35–70 | 327/141 988 | Males: |  |  |  |  |  |
|  |  |  |  | F.35–70 | 528/336 052 | Category 2:<0.8 g/d | 1.09(0.70–1.69) |  |  |  |  |
|  |  |  |  |  |  | Category 3: 0.8–2.3 g/d | 1.17(0.84–1.63) |  |  |  |  |
|  |  |  |  |  |  | Category 4: 2.3–6.2 g/d | 1.17(0.80–1.73) |  |  |  |  |
|  |  |  |  |  |  | Category 5:>6.2 g/d | 1.01(0.67–1.53) |  |  |  |  |
|  |  |  |  |  |  | Females: |  |  |  |  |  |
|  |  |  |  |  |  | Category 2:<0.8 g/d | 1.01(0.72–1.41) |  |  |  |  |
|  |  |  |  |  |  | Category 3: 0.8–2.3 g/d | 1.01(0.77–1.32) |  |  |  |  |
|  |  |  |  |  |  | Category 4: 2.3–6.2 g/d | 0.92(0.70–1.23) |  |  |  |  |
|  |  |  |  |  |  | Category 5:>6.2 g/d | 0.69(0.50–0.95) |  |  |  |  |
| Singh & Fraser | Cohort study | 6 | American | M&F.≥25 | 135/32 051 | Nut consumption: never-1time/week (Ref) |  | Adjusted： age at baseline, sex, BMI, physical activity,  parental history of colon cancer, current smoking, past smoking, alcohol consumption, and aspirin use | Colon cancer | 8 |  |
| 1998 |  |  |  |  |  | 1–4 times/week | 0.67(0.45-0.98) |  |  |  |  |
|  |  |  |  |  |  | >4 times/week | 0.68(0.45-1.04) |  |  |  |  |
| Mills | Cohort study | 6 | American | M.≥25 | 180/14 000 | Current nut intake:<once/week(Ref) |  | Adjusted： age, education, current intakes of meat, poultry, and | Prostate Cancer | 7 |  |
| References | Study type | Follow-up (year) | Country | Age(year) | No .of cases/ no. of overall subjects in CH or no. of controls in CC | Exposure categories (exposure/case assessment) | RR (95%CI) | Monotherapy or Adjusted and Matched/adjusted factors | Outcome investigated | NOS |  |
| 1989 |  |  |  |  |  | 1–4 times/week | 0.86 (0.59–1.24) | fish; intakes of beans, legumes, or peas; intakes of citrus fruit and dried fruit; index of fruit, nuts, and tomatoes |  |  |  |
|  |  |  |  |  |  | ≥5 times/week | 0.79 (0.51–1.22) |  |  |  |  |
| Lee  2018 | CC study | NA | Korea | M. NA | 625/1250 | nut consumption: None(Ref) |  | Adjusted：age, education level, body mass index, alcohol consumption, regular exercise (no, yes), intakes of fruits and vegetables, red meat, calcium, and vitamin D, total energy intake (continuous). | Colorectal cancer | 8 |  |
|  |  |  |  | F. NA | 298/596 | Men |  |  |  |  |  |
|  |  |  |  |  |  | < 1 serving per week | 0.86(0.7-1.06) |  |  |  |  |
|  |  |  |  |  |  | 1–3 servings per week | 0.67(0.46-0.98) |  |  |  |  |
|  |  |  |  |  |  | ≥ 3 servings per week | 0.36(0.24-0.54) |  |  |  |  |
|  |  |  |  |  |  | Women |  |  |  |  |  |
|  |  |  |  |  |  | < 1 serving per week | 0.71(0.52-0.97) |  |  |  |  |
|  |  |  |  |  |  | 1–3 servings per week | 0.31(0.17-0.55) |  |  |  |  |
|  |  |  |  |  |  | ≥ 3 servings per week | 0.21(0.12-0.38) |  |  |  |  |
| Lee  2017 | CC study | NA | American | M&F.35-79 | 1721/1918 | Quintile of total nuts:1: 0 (Ref) |  | Adjusted： age, sex, and cigarette smoking dose, body mass index (BMI), education, cigarette smoking status, and years since last cigarette smoked for former-smokers energy intake. fruits, vegetables, red and processed meat, and alcohol. | Lung cancer | 8 |  |
|  |  |  |  |  |  | 2(1–6 times a year) | 0.92(0.58-1.45) |  |  |  |  |
|  |  |  |  |  |  | 3(7–11 times a year) | 0.76(0.59-0.99) |  |  |  |  |
|  |  |  |  |  |  | 4(1-3times/month | 0.9(0.71-1.14) |  |  |  |  |
|  |  |  |  |  |  | 5 1-6 Times/week | 0.74(0.57-0.95) |  |  |  |  |
| Alejandro D  2015 | CC study | NA | American | F.mean 51 | 97/104 | Peanut intake frequency: low intake (Ref) |  | Adjusted：Smoking, Alcoholism, Diabetes, BMI, Age of first coitus, Pregnancies, Lactation, Hormonal contraceptive, Menarche-menopause, soft drinks, Fruit, Number of pregnancies, Red meat, Beans. | Breast cancer | 8 |  |
|  |  |  |  |  |  | High | 0.33(0.17-0.63) |  |  |  |  |
|  |  |  |  |  |  | Walnut intake frequency: low intake (Ref) |  |  |  |  |  |
|  |  |  |  |  |  | High | 0.45(0.22–0.93) |  |  |  |  |
|  |  |  |  |  |  | Almond intake frequency: low intake (Ref) |  |  |  |  |  |
|  |  |  |  |  |  | High | 0.44(0.21–0.89) |  |  |  |  |
| Liu  2014 | CC study | NA | Canada | F.25-74 | 2865/3299 | Nut intake:<1 time/month (Ref) |  | Adjusted： age, family history of breast cancer in mother and sisters, age at menarche, parity, age at first birth, education, ethnicity, oral contraceptive use, adult BMI, breastfeeding, menopausal status, hormone replacement therapy, and alcohol consumption 2 y before study enrollment | Breast Cancer | 7 |  |
|  |  |  |  |  |  | 1–3/month | 0.86(0.71–1.04) |  |  |  |  |
|  |  |  |  |  |  | 1–6/week | 0.86(0.72–1.04) |  |  |  |  |
|  |  |  |  |  |  | ≥1/day | 0.76(0.61–0.95) |  |  |  |  |
| Jackson  2013 | CC study | NA | Jamaica | M.40–80 | 243/273 | Nut intake: Tertile 1 (Ref) |  | Adjusted： age, family history of prostate cancer, education, BMI, smoking, physical activity, and total energy intake | Prostate cancer | 7 |  |
|  |  |  |  |  |  | Tertile 2 | 1.31(0.79-2.18) |  |  |  |  |
| References | Study type | Follow-up (year) | Country | Age(year) | No .of cases/ no. of overall subjects in CH or no. of controls in CC | Exposure categories (exposure/case assessment) | RR (95%CI) | Monotherapy or Adjusted and Matched/adjusted factors | Outcome investigated | NOS |  |
|  |  |  |  |  |  | Tertile 3 | 0.81 (0.46–1.42) |  |  |  |  |
| Moller  2013 | CC study | NA | Sweden | M.35–79 | 1482/1108 | low intake: <75th centile intake among controls |  | Adjusted： age, region, education, smoking, BMI, energy intake, physical activity, diabetes, and family history of PC | Prostate cancer | 7 |  |
|  |  |  |  |  |  | High intake: >75th centile intake among controls | 1.03(0.84–1.25) |  |  |  |  |
| Takayama  2013 | CC study | NA | Japan | F.mean 54 | 161/380 | Peanut intake frequency: no intake (Ref)  1–3 times/month  ≥1–2 times/week | 1.22(0.76-1.95) | Adjusted： age, BMI, diabetes history, and hypertension  history | Endometrial cancer | 6 |  |
|  |  |  |  |  |  |  | 0.48(0.27-0.86) |  |  |  |  |
|  |  |  |  |  |  | Nut intake:≤ 0.53 g/1000 kcal (Ref) |  |  |  |  |  |
|  |  |  |  |  |  | 0.54–1.16 g/1000 kcal | 0.93(0.53–1.54) |  |  |  |  |
|  |  |  |  |  |  | 1.17–2.25 g/1000 kcal | 1.33(0.77–2.31 |  |  |  |  |
|  |  |  |  |  |  | ≥2.26 g/1000 kcal | 0.46(0.25–0.86) |  |  |  |  |
| Ibiebele | CC study | NA | Australia | F.18-79 | 1366/1414 | Omega-6 fatty acid (g) from nuts: |  | Adjusted: age, education, BMI, smoking status, oral contraceptive use, parity, menopausal status, hormonal replacement therapy, total fat intake, and total energy intake | Ovarian cancer | 8 |  |
| 2012 |  |  |  |  |  | 0.13 (0.0–0.29)(Ref) |  |  |  |  |  |
|  |  |  |  |  |  | 0.45 (0.29–0.68) | 0.83 (0.67–1.03) |  |  |  |  |
|  |  |  |  |  |  | 1.48 (0.73–2.59) | 0.88 (0.71–1.09) |  |  |  |  |
|  |  |  |  |  |  | 3.35 (2.59–25.9) | 0.72 (0.57–0.92) |  |  |  |  |
| Wang  2012 | CC study | NA | China | M&F.30-79 | 257/514 | Nut intake: Tertile 1(Ref) |  | Adjusted： sex, area, education, smoking, alcohol consumption, family history, total vegetable intake, total fruit intake, pickled food intake, soy products intake, total energy intake, meat intake, and Helicobacter pylori | Gastric cancer | 8 |  |
|  |  |  |  |  |  | Tertile 2 | 0.9(0.2–2.7) |  |  |  |  |
|  |  |  |  |  |  | Tertile 3 | 0.9(0.3–3.3) |  |  |  |  |
| Raimondi  2010 | CC study | NA | Canada | M.35–84 | 197/197 | Nut consumption: 0 g/d (Ref) |  | Adjusted： age, place of residence, family history of prostate cancer, total energy intake | Prostate cancer | 7 |  |
|  |  |  |  |  |  | 0.1–1.2 g/d | 0.91(0.47–1.76) |  |  |  |  |
|  |  |  |  |  |  | 1.3–3.0 g/d | 1.10(0.52–2.29) |  |  |  |  |
|  |  |  |  |  |  | >3.0 g/d | 0.43(0.22–0.85) |  |  |  |  |
| Soliman  2010 | CC study | NA | Egypt | M&F.NA | 150/150 | Peanut consumption: no intake (Ref) |  | Adjusted： age, sex, viral infection | Liver cancer | 7 |  |
|  |  |  |  |  |  | 1–2 times/y | 0.64(0.16-2.57) |  |  |  |  |
|  |  |  |  |  |  | >2 times/y | 0.59(0.13-2.61) |  |  |  |  |
| Pan | CC study | NA | Canada | F.mean 55 | 442/2135 | Nut products intake: First quartile (Ref) |  | Adjusted： age, sex, 10-y age group, province of residence, | Ovarian cancer | 8 |  |
| References | Study type | Follow-up (year) | Country | Age(year) | No .of cases/ no. of overall subjects in CH or no. of controls in CC | Exposure categories (exposure/case assessment) | RR (95%CI) | Monotherapy or Adjusted and Matched/adjusted factors | Outcome investigated | NOS |  |
| 2004 |  |  |  |  |  | Second quartile | 1.22(0.89–1.67) | education, alcohol consumption, cigarette pack-years, BMI, total caloric intake, recreational physical activity, number of live births, menstruation years, and menopause status |  |  |  |
|  |  |  |  |  |  | Third quartile | 1.04(0.75–1.45) |  |  |  |  |
|  |  |  |  |  |  | Fourth quartile | 1.13(0.82–1.55) |  |  |  |  |
| Yu  2002 | CC study | NA | China | M&F.25-79 | 248/248 | Peanut intake:<3 times/week (Ref)  ≥3 times/week | 0.66(0.32–1.36) | Adjusted： Sex, age, residence, HBV infection | Liver cancer | 8 |  |
|  |  |  |  |  |  |  |  |  |  |  |  |
| Hoshiyama  1992 | CC study | NA | Japan | M&F.NA | 294/294 | Nut intake: never (Ref) |  | Adjusted： sex, age, area, smoking status, intakes of salty foods, rice, miso soup, boiled fish, pickled vegetables, seaweed, soybean products, fruits, green–yellow vegetables, raw vegetables | Stomach cancer | 7 |  |
|  |  |  |  |  |  | ≥2 times/month | 0.7 (0.4–1.3) |  |  |  |  |
|  |  |  |  |  |  | ≥3 times/month | 0.6 (0.3–1.0) |  |  |  |  |
| Chen | CC study | NA | China | M&F.NA | 200/200 | Peanut consumption:<1 meal/week(Ref) |  | Adjusted： age, sex, ethnic group, residential area | Liver cancer | 7 |  |
| 1991 |  |  |  |  |  | ≥1 meal/week | 1.44(0.94–2.21) |  |  |  |  |
| Trichopoulos  1985 | CC study | NA | Greece | M&F.NA | 110/100 | Nut consumption frequency: 0 (Ref) |  | Adjusted：sex, age, area, smoking status | Stomach cancer | 6 |  |
|  |  |  |  |  |  | 2 times/month | 0.64(0.30–1.39) |  |  |  |  |
|  |  |  |  |  |  | 4 times/month | 0.45(0.20–0.99) |  |  |  |  |
|  |  |  |  |  |  | 10 times/month | 1.96(0.75–5.17) |  |  |  |  |
|  |  |  |  |  |  | 30 times/month | 1.88(1.22–2.89) |  |  |  |  |

Abbreviations: RR, relative risk; CI, confidence interval, CH, cohort study; CC, case-control study; M, male; F, female; Ref, reference; NO. of cases/subjects, number of cases/subjects; NOS, Newcastle–Ottawa scale
