# Supplementary material for: Meta-analysis of the association between nut consumption and the risks of cancer incidence and cancer-specific mortality
Source: Aging (Albany NY). 2020 Jun 2;12(11):10772–94. doi: 10.18632/aging.103292 (PMC7346045; doi:10.18632/aging.103292)
Supplement: Supplementary Table 2 [file aging-12-103292-s001..docx]

**Supplementary Table 2. Characteristics of included studies on nut intake and cancer-specific mortality in the meta-analysis.**

| References | Country | | Study type and Follow-up (year) | | Age(year) | No. of cases /no. of subjects or person years | Exposure categories (exposure/case assessment) | OR (95%CI) | | Monotherapy or Adjusted and Matched/adjusted factors | NOS |
| --- | --- | --- | --- | --- | --- | --- | --- | --- | --- | --- | --- |
| Amba | America | | CH 15.5 | | M&F.50-71 | 26685/374101 | Total nut intake(g/1000kcal):C1 0 (Ref) | | | Adjusted: age, sex, BMI level of education, race, self-reported health condition, smoking status, total energy consumption, alcohol consumption, vitamin consumption physical activity and food groups including white meat, red meat (grams per day), whole grain, vegetable and fruit | 9 |
| 2019 |  | |  | |  |  | C2: 0.05-0.17 | 0.91(0.87-0.95) | |  |  |
|  |  | |  | |  |  | C3: 0.36-0.68 | 0.88(0.83-0.92) | |  |  |
|  |  | |  | |  |  | C4: 1.34-4.14 | 0.88(0.84-0.92) | |  |  |
| Liu | America | | CH 34 | | M&F.30-55 | 1297/16217 | Total Nut:<1 serving/month (Ref) | | | Adjusted: age, diabetes duration (years), sex, Caucasian (yes/no), BMI at diabetes diagnosis, physical activity, smoking status, alcohol consumption, family history of MI or cancer (yes/no), current aspirin use (yes/no), presence of hypertension (yes/no), use of lipid-lowering medication (yes/no),  diabetes medication use, and intake of total energy, red or processed meat, fruits, and vegetables | 7 |
| 2018 |  | |  | |  |  | <1 serving/week | 0.93(0.79-1.1) | |  |  |
|  |  | | |  |  |  | 1 serving/week | 0.91(0.76-1.09) | |  |  |
|  |  | | |  |  |  | 2-4 servings/week | 0.89(0.75-1.07) | |  |  |
|  |  | | |  |  |  | ≥5 servings/week | 0.84(0.67-1.06) | |  |  |
| Eslamparast | Iran | | | CH 7 | M&F.≥40 | 887/349677 | Nut Consumption: Never (Ref) |  | | Adjusted: age at enrolment, sex, BMI, level of education, place of residence, smoking status, opium and alcohol consumption, physical activity level, wealth score (WS), diabetes, hypertension, total energy intake, main food groups, magnesium (Mg), zinc (Zn) and copper | 7 |
| 2016 |  | | |  |  |  | <1 serving per week | 0.96(0.82-1.11) | |  |  |
|  |  | | |  |  |  | 1 to < 3 servings per week | 0.84(0.65-1.07) | |  |  |
|  |  | | |  |  |  | ≥3 servings per week | 0.62(0.38-1.01) | |  |  |
| Brandt | Netherlands | | | CH 10 | M&F.55-69 | 3917/120852 | Total nut intake(g/day):0 (Ref) |  | | Adjusted: : age at baseline, sex, cigarette smoking, number of cigarettes smoked per day, and years of smoking, history of physician-diagnosed hypertension (no, yes) and diabetes (no, yes), body height, BMI, non-occupational physical activity, highest level of education, intake of alcohol, vegetables and fruit, energy, use of nutritional supplements (no, yes), and, in women, postmenopausal HRT | 7 |
| 2015 |  | | |  |  |  | 0.1–<5 g/d | 0.92(0.81-1.05) | |  |  |
|  |  | | |  |  |  | 5–<10 g/d | 0.82(0.68-0.98) | |  |  |
|  |  | | |  |  |  | 10+g/d | 0.79(0.67-0.93) | |  |  |
| Bonaccio | Italy | | | CH 4.3 | M&F.55-80 | 122/19386 | Nut Consumption: no intake (Ref) |  | | Adjusted: age, sex, educational level (low/high), smoking, leisure-time physical activity, BMI, energy intake. | 6 |
| 2015 |  | | |  |  |  | intake | 0.64(0.44-0.94） | |  |  |
| Hshieh | America | | | CH 9.6 | M.NA | 868/20742 | Nut Consumption:1 serving/month (Ref) | | | Adjusted: age, BMI, alcohol consumption, smoking, exercise, calories, saturated fat consumption, fruit/vegetable consumption, red meat consumption, prevalent diabetes, and hypertension. | 7 |
| 2015 |  | | |  |  |  | 1–3 servings/month | 0.91(0.77-1.08) | |  |  |
|  |  | | |  |  |  | 1 serving/week | 0.88(0.72-1.07) | |  |  |
|  |  | | |  |  |  | 2–4 servings/week | | 0.87(0.68-1.09) |  |  |
|  |  | | |  |  |  | ≥5 servings/week | | 0.87(0.66-1.15) |  |  |
| Luu | US-African | | | CH 5.4 | M&F.40-79 | 1053/48347 | Total Nut(g/d):<0.95 (Ref) | |  | Adjusted: age, sex, education, occupation, household income (SMHS) or income per capita (SWHS), smoking status, alcohol consumption (ever/never), BMI, physical activity, regular tea consumption, Charlson Comorbidity Index, total energy intake, red meat intake, chicken/duck intake, seafood intake, vegetable intake, and fruit intake.  Adjusted: age, sex, education, occupation, household income (SMHS) or income per capita, smoking status, alcohol consumption (ever/never), BMI, physical activity, regular tea consumption, Charlson Comorbidity Index, total energy intake, red meat intake, chicken/duck intake, seafood intake, vegetable intake, and fruit intake.  Adjusted: age, sex, education, occupation, household income (SMHS) or income per capita (SWHS), smoking status, alcohol consumption, BMI, physical activity, regular tea consumption, Charlson Comorbidity Index, total energy intake, red meat intake, chicken/duck intake, seafood intake, vegetable intake, and fruit intake. | 7 |
| 2015 |  | | |  |  |  | 0.95 to<3.08 | | 0.89(0.73-1.07) |  |  |
|  |  | | |  |  |  | 3.08 to <7.30 | | 0.85(0.69-1.05) |  |  |
|  |  | | |  |  |  | 7.30 to <18.45 | | 0.91(0.74-1.11) |  |  |
|  |  | | |  |  |  | ≥18.45 | | 0.74(0.6-0.92) |  |  |
|  | US-European | | | CH 6.5 | M&F.40-79 | 499/23417 | Total Nut(g/d):<0.95 (Ref) | |  |  | 7 |
|  |  | | |  |  |  | 0.95 to<3.08 | | 0.98(0.72-1.32) |  |  |
|  |  | | |  |  |  | 3.08 to <7.30 | | 0.89(0.65-1.21) |  |  |
|  |  | | |  |  |  | 7.30 to <18.45 | | 0.68(0.48-0.84) |  |  |
|  |  | | |  |  |  | ≥18.45 | | 0.93(0.68-1.92) |  |  |
|  | Asian | | | CH 12.2 | M&F.40-70 | 3532/134265 | Total Nut(g/d):<0.14 (Ref) | |  |  | 8 |
|  |  | | |  |  |  | 0.14 to <0.72 | | 0.88(0.78-0.98) |  |  |
|  |  | | |  |  |  | 0.72 to <1.45 | | 0.91(0.82-1.1) |  |  |
|  |  | | |  |  |  | 1.45 to <2.54 | | 0.96(0.87-1.05) |  |  |
|  |  | | |  |  |  | ≥2.54 | | 0.95(0.86-1.06) |  |  |
| Bao | America | | | CH 24 | M&F. NA | 10293/118926 | Nut Consumption: Never (Ref) | |  | Adjusted：age; race; body-mass index; level of physical activity; smoking， | 6 |
| References | Country | | Study type and Follow-up (year) | | Age(year) | No. of cases /no. of subjects or person years | Exposure categories (exposure/case assessment ) | | OR (95%CI) | Monotherapy or Adjusted and Matched/adjusted factors | NOS |
| 2013 | |  |  | |  |  | <Once/week | | 0.93(0.88-0.98) | current multivitamin use, and current aspirin use; myocardial infarction, or cancer; a history of diabetes mellitus, hypertension, intake of total energy, alcohol, red or processed meat, fruits, and vegetables, menopausal status and hormone use. |  |
|  | |  |  | |  |  | Once per week | | 0.93(0.87-1.00) |  |  |
|  | |  |  | |  |  | 2–4 Times/week | | 0.92(0.85-0.98 |  |  |
|  | |  |  | |  |  | ≥5 Times/week | | 0.89(0.81-0.99) |  |  |
| Guasch-Ferré | | Spain | CH 4.8 | | M&F.55-80 | 130/31077 | Total nut intake: 0 (Ref) | |  | Adjusted: age, sex, BMI smoking status, educational level, leisure time physical activity in MET-min/day, history of diabetes, history of hypercholesterolemia, use of oral antidiabetic medication, use of antihypertensive medication, use of statins, and total energy intake | 8 |
| 2013 | |  |  | |  |  | 1 to 3 servings/week | | 0.79(0.52-1.2) |  |  |
|  | |  |  | |  |  | >3 servings/week | | 0.6(0.37-0.98) |  |  |
|  | |  | |  |  |  |  | |  |  |  |

Abbreviations: OR, odds ratio; CI, confidence interval, M, male; F, female; Ref, reference; NO. of cases/subjects, number of cases/subjects; NOS, Newcastle–Ottawa scale; CH: cohort study.
